# Supplementary material for: Peptide-oligourea hybrids analogue of GLP-1 with improved action in vivo
Source: Nat Commun. 2019 Feb 25;10:924. doi: 10.1038/s41467-019-08793-y (PMC6389962; doi:10.1038/s41467-019-08793-y)
Supplement: Supplementary file 1 — Supplementary Information [file 41467_2019_8793_MOESM1_ESM.pdf]

Supplementary information

Peptide-Oligourea Hybrids Analogue of GLP-1 with Improved Action in Vivo

Juliette Fremaux, Claire Venin, Laura Mauran, Robert H. Zimmer, Gilles Guichard, Sébastien R. Goudreau

Supplementary Figures

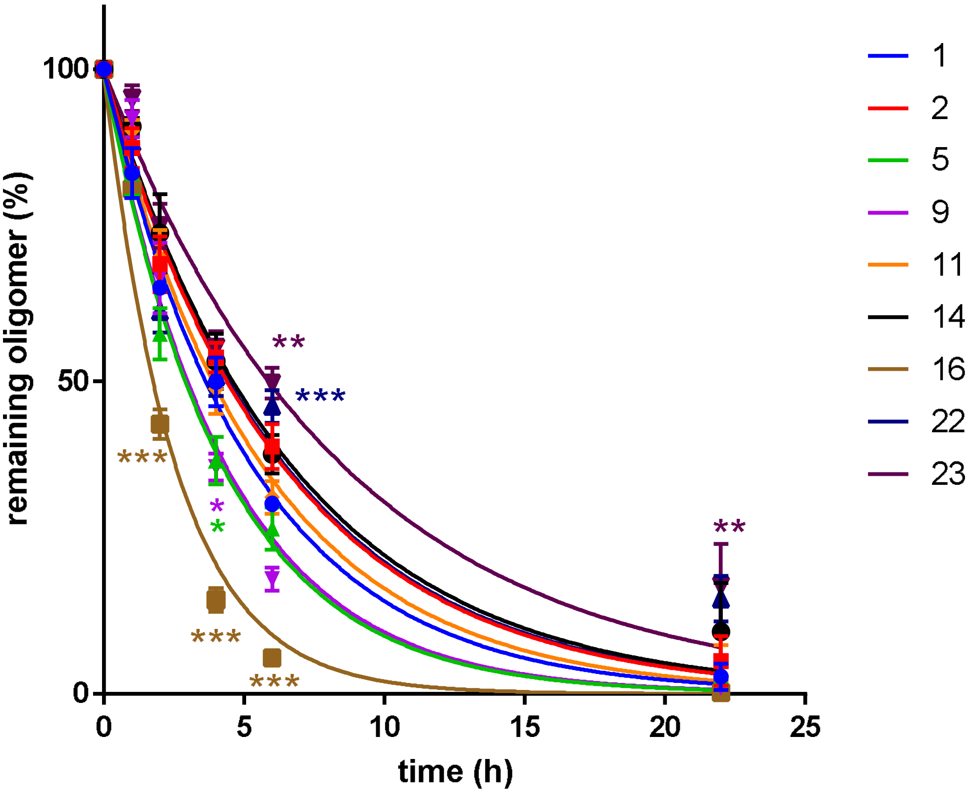

Supplementary Figure 1 : NEP 24.11 Degradation Assay. Stability of selected GLP-1 analogues in solution with NEP 24.11. Data are mean ± SEM, n = 3. Statistic by two-way anova and Bonferroni post-test: *p<0.05, **p<0.01, ***p<0.001, comparing peptide **1** to hybrids. Source data are provided as a Source Data file (Table 2 tab).

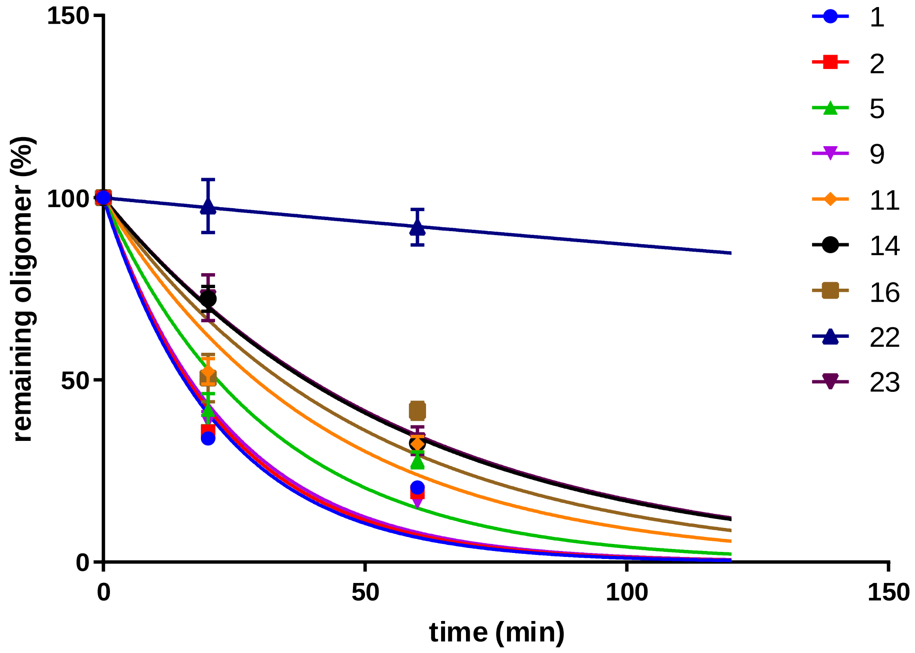

Supplementary Figure 2 : **Mouse plasma degradation assay.** Stability of selected GLP-1 analogues in mouse plasma. Data are mean ± SEM, n = 4. Source data are provided as a Source Data file (Table 2 tab).

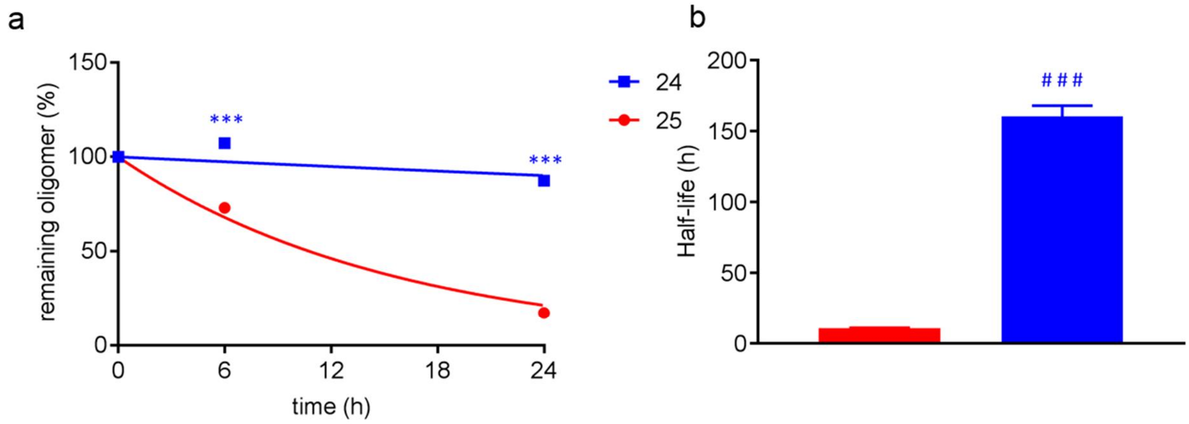

c

Sequence	Compound	Half-life (h)	SEM (h)
**24**	semaglutide-[$A^uA^uA^u$]$^{28\text{-}31}$	160	4.38
**25**	semaglutide	11	0.03

Supplementary Figure 3. Mouse plasma degradation assay on semaglutide and semaglutide analogue 24. Stability of semaglutide and analogue **24** in mouse plasma. (a) Chart of remaining oligomers versus time. (b) Bar chart of half-lives. (c) Table of results. Data are mean ± SEM, n = 3. Statistic by two-way anova and Bonferroni post-test: ***p<0.001, comparing semaglutide to **24**; two-way t-test: ###p<0.001, comparing semaglutide to **24**. Source data are provided as a Source Data file (Supp Fig. 3 tab).

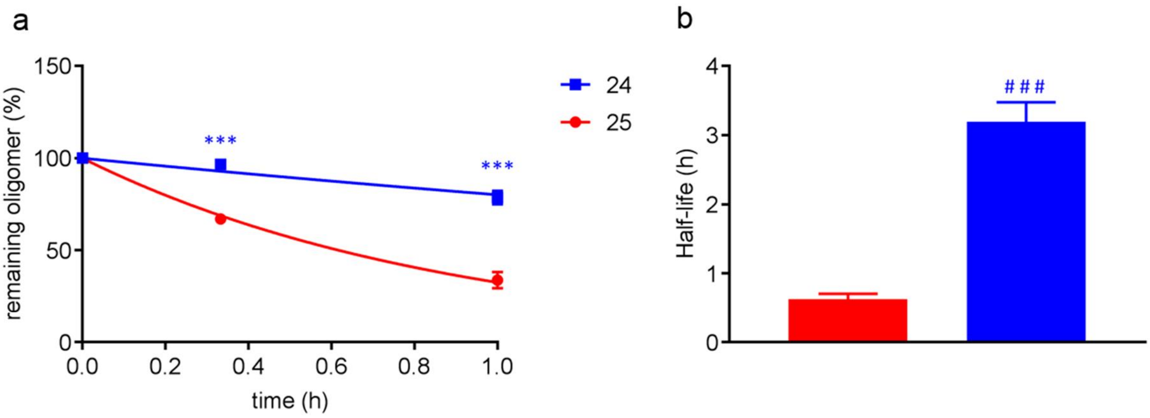

c

Sequence	Compound	Half-life (h)	SEM (h)
**24**	semaglutide-[A^{u}A^{u}A^u]$^{28-31}$	3.20	0.28
**25**	semaglutide	0.63	0.08

Supplementary Figure 4. Pancreatin degradation assay. Stability of semaglutide **25** and analogue **24** in pancreatin. (a) Chart of remaining oligomers versus time. (b) Bar chart of half-lives. (c) Table of results. Data are mean ± SEM, n = 3. Statistic by two-way anova and Bonferroni post-test: ***p<0.001, comparing semaglutide to **24**; two-way t-test: ###p<0.001, comparing semaglutide to **24**. Source data are provided as a Source Data file (Fig. 6b tab).

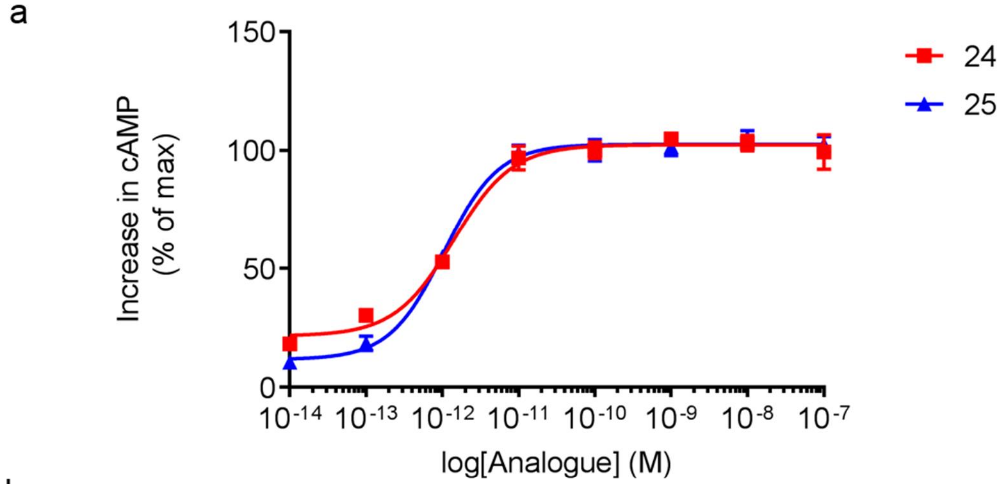

b

Sequence	Compound	EC_{50} (pM)	SEM (pM)
**24**	semaglutide-[A^{u}A^{u}A^u]$^{28-31}$	1.07	0.03
**25**	semaglutide	1.38	0.08

Supplementary Figure 5. ***In vitro*** **characterization of semaglutide and analogue 24.** (a) Evaluation of the agonist activity (EC_{50}) of hybrid **24** and semaglutide (**25**) at the human GLP-1 receptor exogenously expressed in HEK293T cells, was determined by measuring their effects on cAMP production using the HTRF detection method. (b) Table of results. Data are mean ± SEM, n = 4. Source data are provided as a Source Data file (Supp Fig. 5 tab).

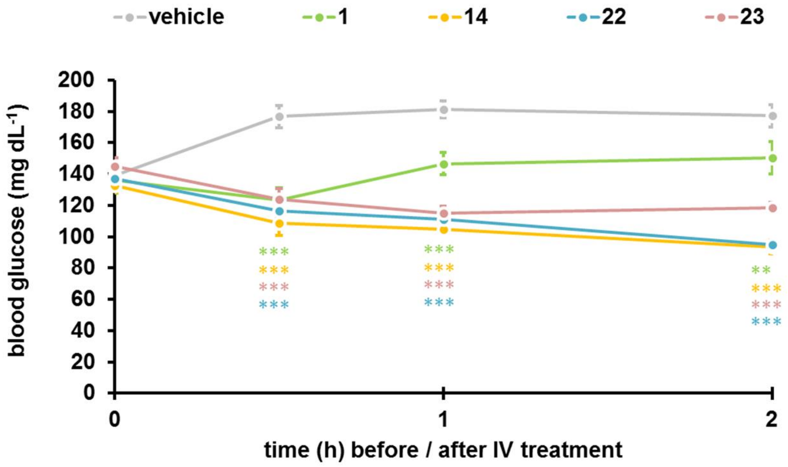

Supplementary Figure 6. Blood glucose measurements prior to the 2 h IPGTT. Blood glucose concentration before and after dosing in fasted normal mice (C57BL/6J, male, 20-25 g) within the 2 h prior to the IPGTT in the 2 h IPGTT experience. Dosage: 200 µg kg^{-1} (~50 nmol kg^{-1}) *i.v.* Formulation: 20 µg mL^{-1} in PBS 1X. Data are mean ± SEM, n = 6. Statistic by two-way anova and Bonferroni post-test: *p<0.05, **p<0.01, ***p<0.001, comparing vehicle to oligomers. Source data are provided as a Source Data file (Fig. 4c tab).

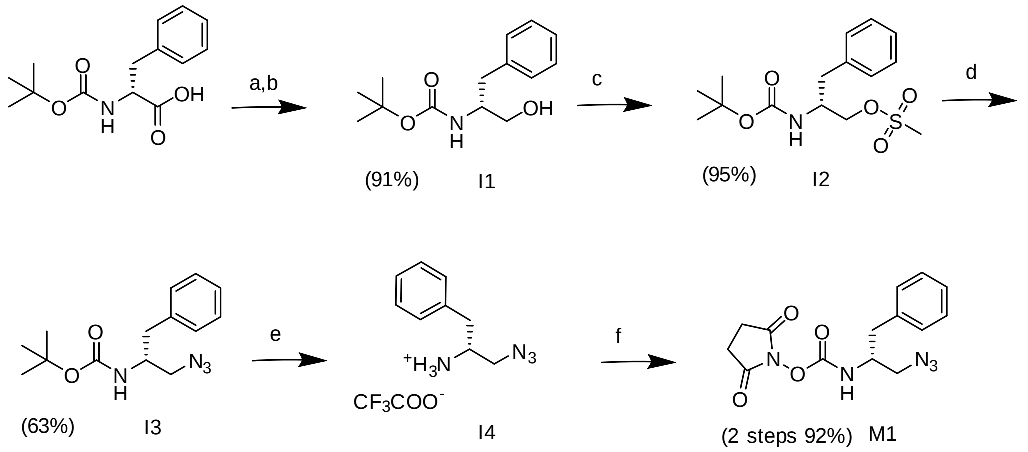

a: iBuOCOCl, NMM, THF, -10°C; b: $NaBH_4$, THF, H_2O, 0°C; c: $MeSO_2Cl$, Et_3N, DCM, 0°C; d: NaN_3, DMF, 80°C; e: TFA; f: DIEA, DSC, DCM, 0°C

Supplementary Figure 7 : Synthesis scheme of monomer M1. Synthesis of monomer **M1** in 6 steps from Boc-*D*-Phe-OH. **M1**: 2,5-dioxopyrrolidin-1-yl (R)-(1-azido-3-phenylpropan-2-yl)carbamate ; (N_3-$F^{u\alpha}$-OSu).

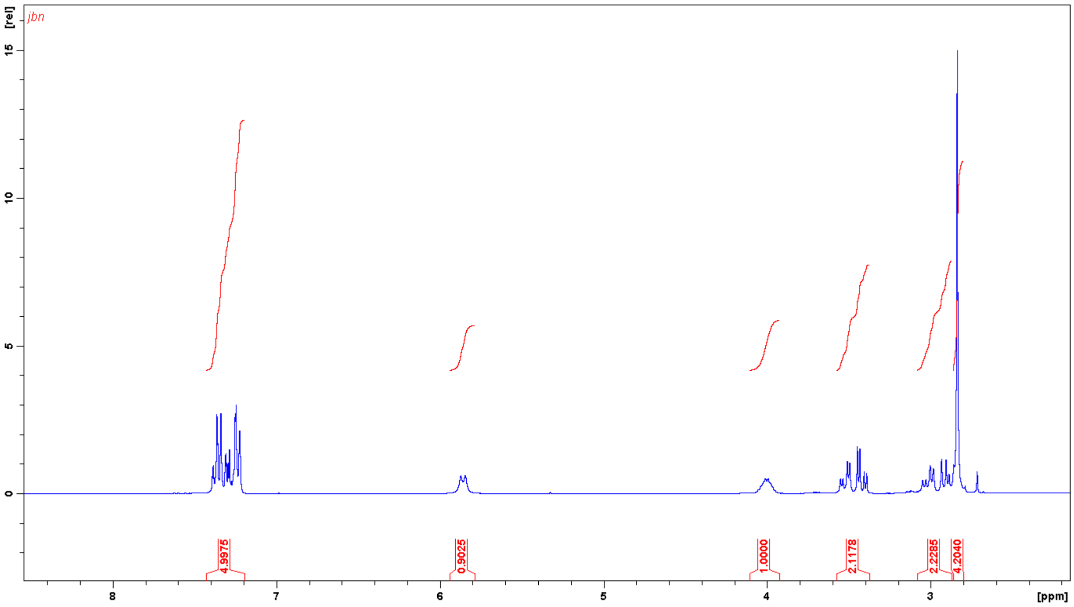

Supplementary Figure 8. ^{1}H NMR of M1. ^{1}H NMR of monomer **M1** in $CDCl_3$ (300 MHz).

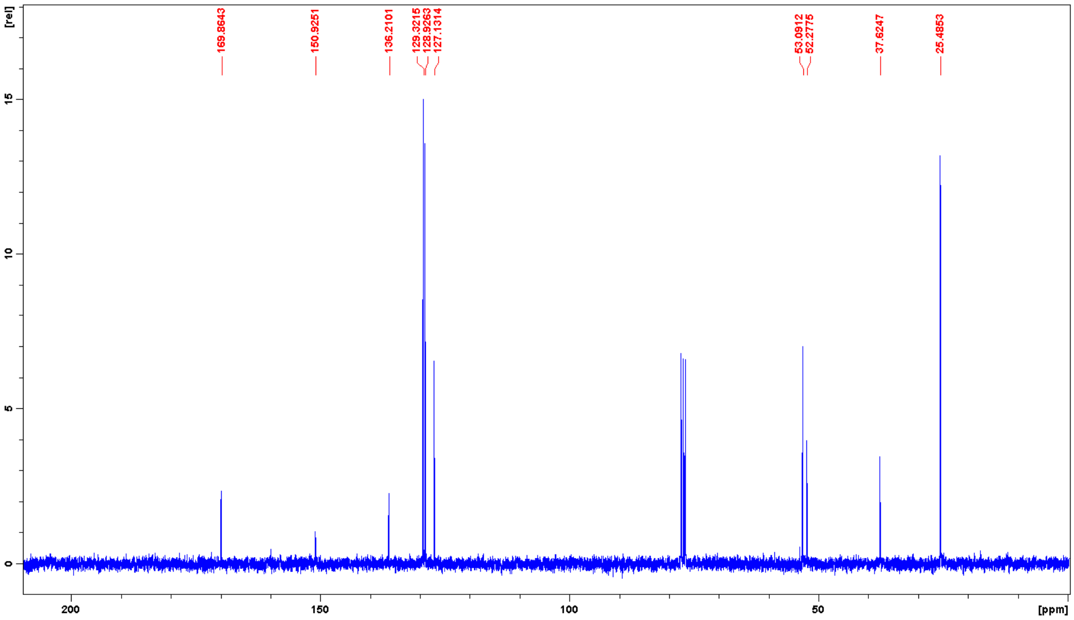

Supplementary Figure 9. ^{13}C NMR of M1. ^{13}C NMR of monomer **M1** in $CDCl_3$ (75 MHz).

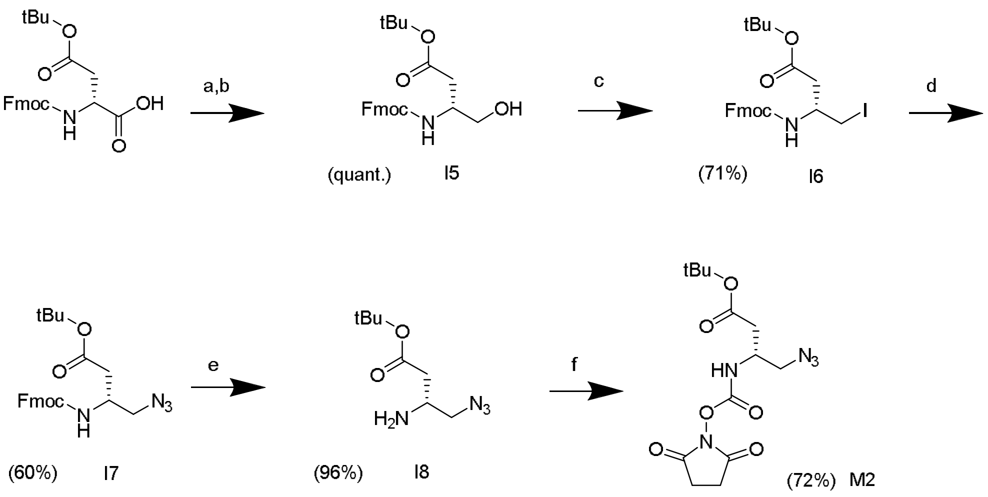

a: iBuOCOCl, NMM, THF, -10°C; b: $NaBH_4$, THF, H_2O, 0°C; c: PPh_3, Imidazole, I_2, DCM; d: NaN_3, DMF; e: Piperidine, THF; f: DSC, EtOAc, 0°C

Supplementary Figure 10 : Synthesis scheme of monomer M2. Synthesis of monomer **M2** in 6 steps from Fmoc-*D*-Asp(*t*Bu)-OH. **M2**: *tert*-butyl (R)-4-azido-3-((((2,5-dioxopyrrolidin-1-yl)oxy)carbonyl)amino)butanoate; (N_3-$D^{u\alpha}$(*t*Bu)-OSu)

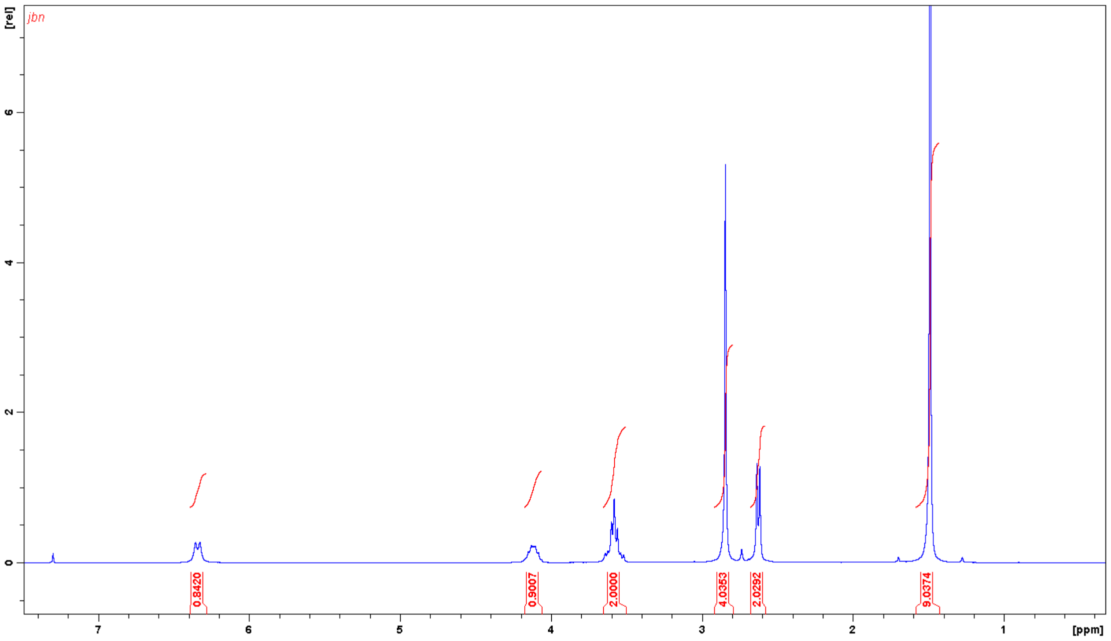

Supplementary Figure 11. ^{1}H NMR of M2. ^{1}H NMR of monomer **M2** in $CDCl_3$ (300 MHz).

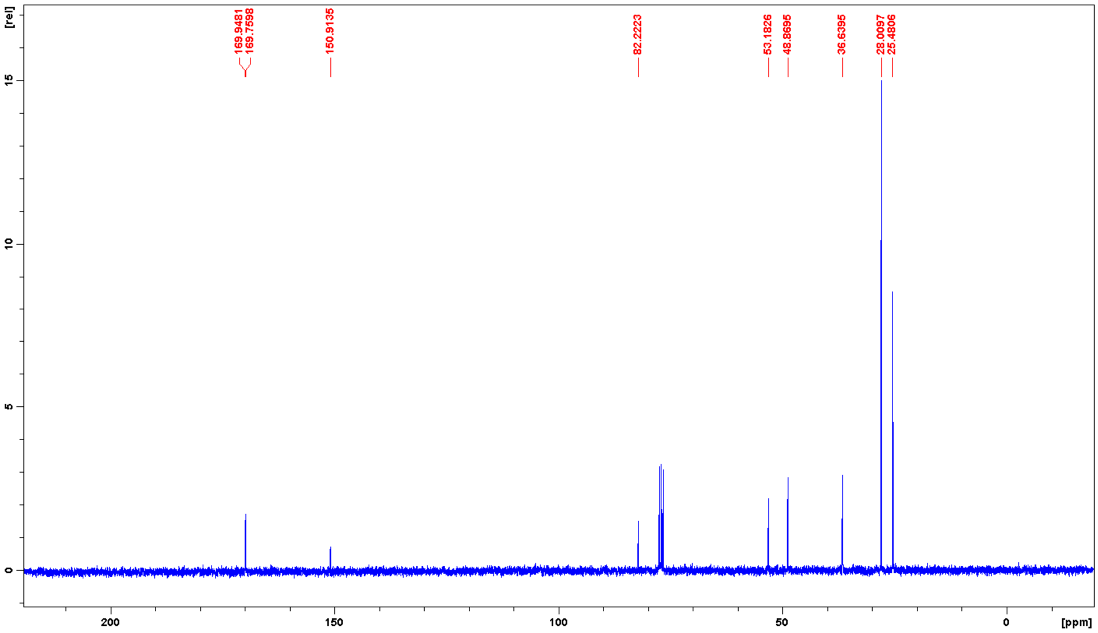

Supplementary Figure 12. ^{13}C NMR of M2. ^{13}C NMR of monomer **M2** in $CDCl_3$ (75 MHz).

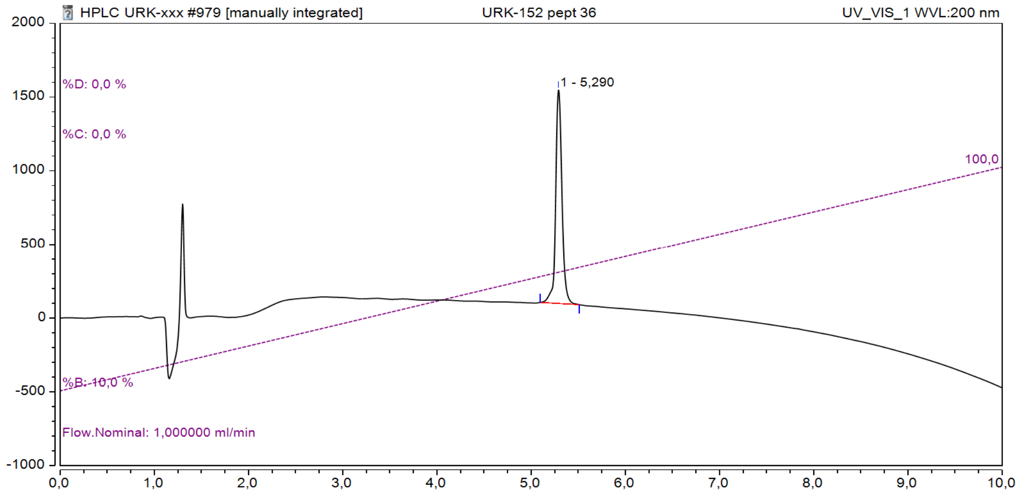

Supplementary Figure 13. HPLC profile of peptide 1. Characterization of peptide **1** by HPLC (10-100%; CH_3CN 0.1 % TFA in H_2O 0.1% TFA, 10 min, C18).

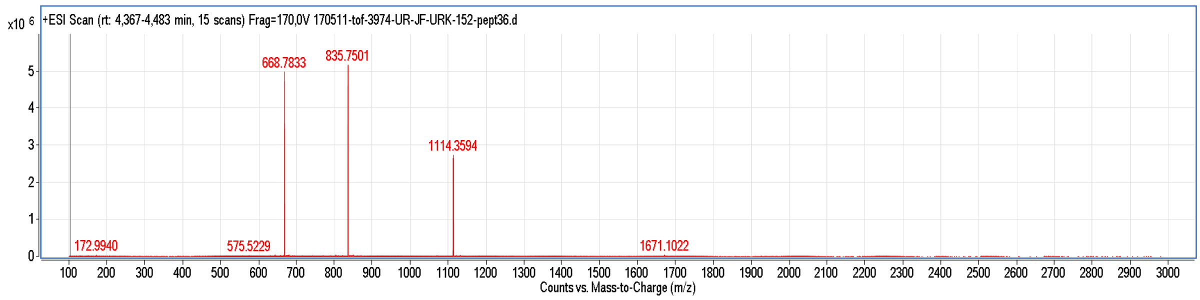

Supplementary Figure 14. LC-MS spectrum of peptide 1. Characterization of peptide **1** by LC-MS. The experiment was performed on UHPLC (Agilent 1290 Infinity) coupled to an ESI -MS Tof (Agilent 6230 ESI) with the dual-ESI source operated in positive ion mode. (Gradient: 10-100%; CH_3CN 0.1 % Formic acid in H_2O 0.1% formic acid, 6 min, C18).

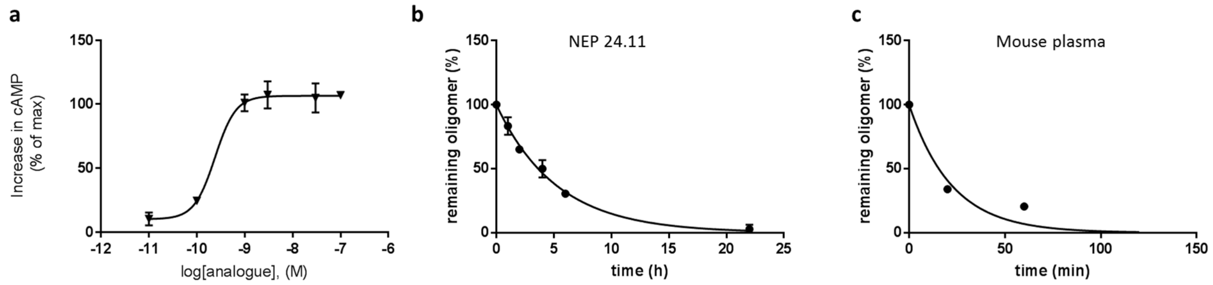

Supplementary Figure 15. *In vitro* pharmacology characterization. (a) Concentration-response curve for EC_{50} determination of peptide **1**. Evaluation of the agonist activity at the mouse GLP-1 receptor endogenously expressed in βTC6 cells, was determined by measuring their effects on cAMP production using HTRF detection method. The data are mean ±SEM of a typical experiment performed twice. (b) Enzymatic degradation (NEP 24.11). The data are mean ±SEM of a typical experiment performed 3 times. (c) Mouse plasma degradation. The data are mean ±SEM of a typical experiment performed 3 times. Source data are provided as a Source Data file ((a): Table 1, Fig. 2 tab. (b-c): Table 2 tab)..

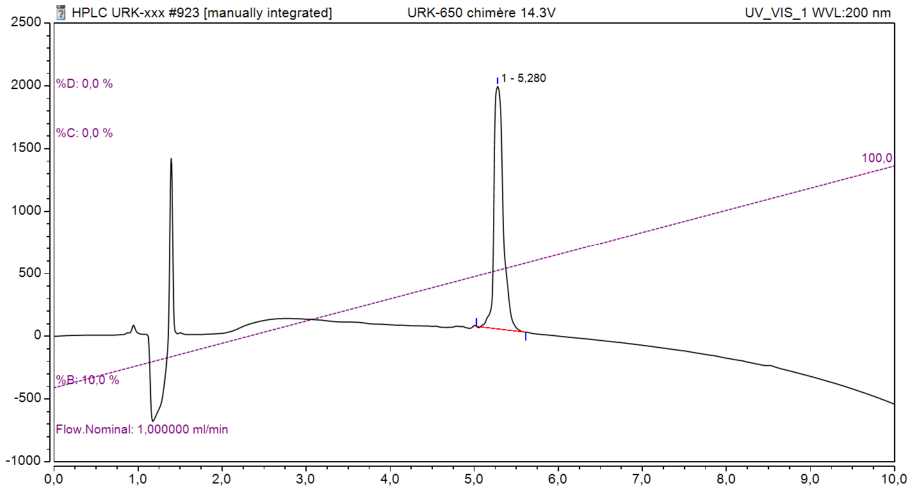

Supplementary Figure 16. HPLC profile. Oligomer **2** (10-100%; CH_3CN 0.1 % TFA in H_2O 0.1% TFA, 10 min, C18)

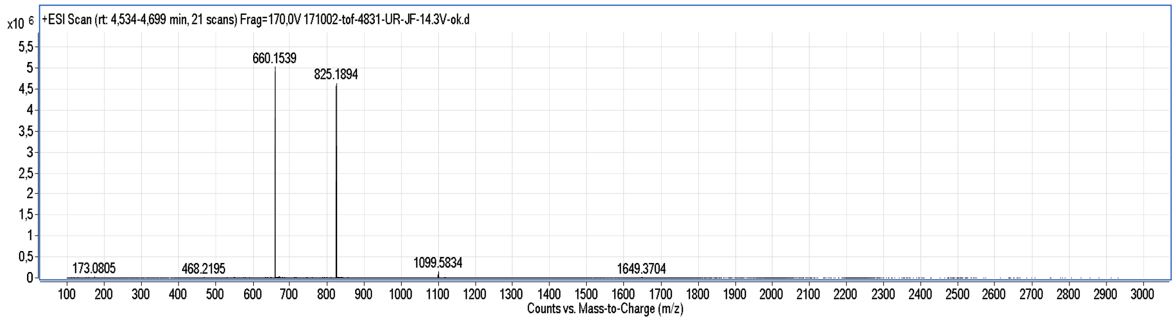

Supplementary Figure 17. LC-MS spectrum of Oligomer 2. Characterization of oligomer **2** by LC-MS. The experiment was performed on UHPLC (Agilent 1290 Infinity) coupled to a ESI -MS Tof (Agilent 6230 ESI) with the dual-ESI source operated in positive ion mode. (Gradient: 10-100%; CH_3CN 0.1 % Formic acid in H_2O 0.1% formic acid, 6 min, C18).

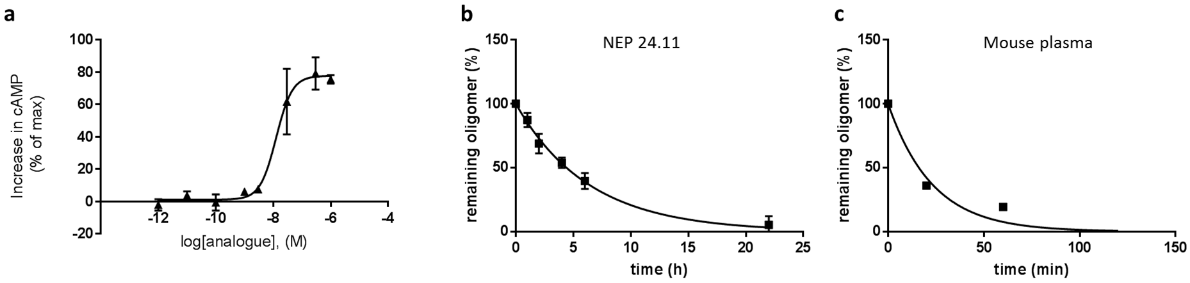

Supplementary Figure 18. In vitro pharmacology characterization. (a) Concentration-response curve for EC_{50} determination of oligomer **2**. Evaluation of the agonist activity at the mouse GLP-1 receptor endogenously expressed in βTC6 cells, was determined by measuring their effects on cAMP production using HTRF detection method. The data are mean ±SEM of a typical experiment performed twice. (b) Enzymatic degradation (NEP 24.11). The data are mean ±SEM of a typical experiment performed 3 times. (c) Mouse plasma degradation. The data are mean ±SEM of a typical experiment performed 3 times. Source data are provided as a Source Data file ((a): Table 1, Fig. 2 tab. (b-c): Table 2 tab)..

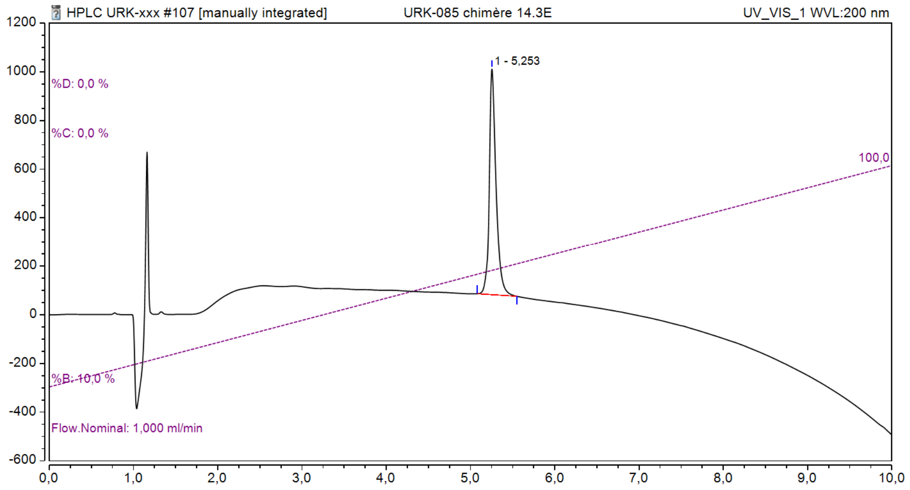

Supplementary Figure 19. HPLC profile. Oligomer **3** (10-100%; CH_3CN 0.1 % TFA in H_2O 0.1% TFA, 10 min, C18)

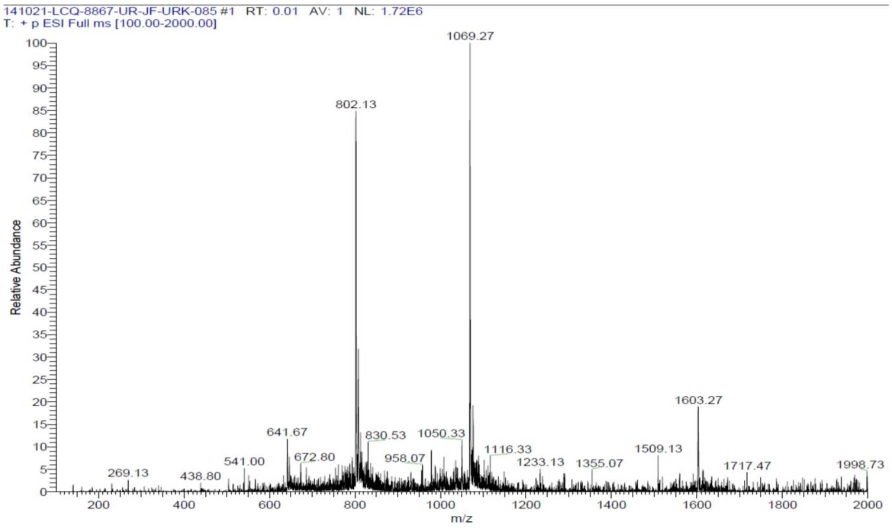

Supplementary Figure 20. Electrospray ionization mass spectrum (ESI-MS) of oligomer 3. The experiment was performed on an Agilent 6560 DTIMS-Q-TOF spectrometer, with the dual-ESI source operated in positive ion mode.

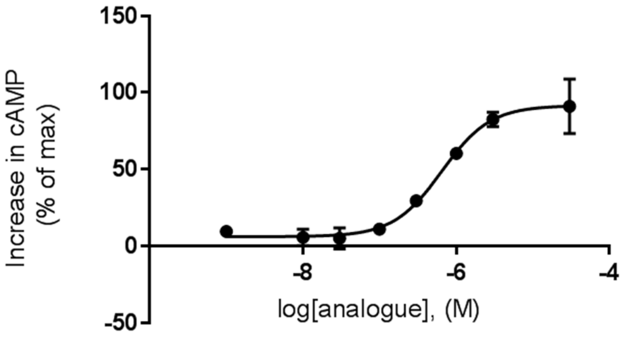

Supplementary Figure 21. In vitro pharmacology characterization. Concentration-response curve for EC_{50} determination of oligomer **3**. Evaluation of the agonist activity at the mouse GLP-1 receptor endogenously expressed in βTC6 cells, was determined by measuring their effects on cAMP production using HTRF detection method. The data are mean ±SEM of a typical experiment performed twice. Source data are provided as a Source Data file (Table 1, Fig. 2 tab).

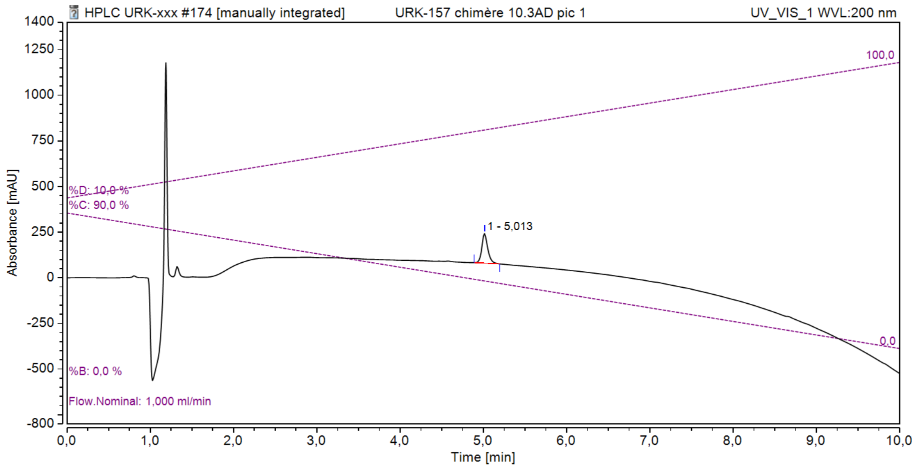

Supplementary Figure 22. HPLC profile. Oligomer **4** (10-100%; CH_3CN 0.1 % TFA in H_2O 0.1% TFA, 10 min, C18)

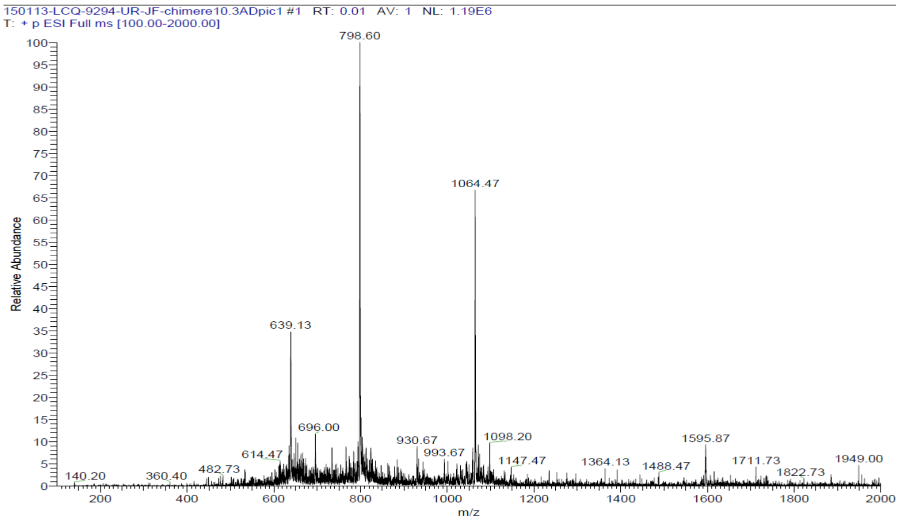

Supplementary Figure 23. Electrospray ionization mass spectrum (ESI-MS) of oligomer 4. The experiments was performed on an Agilent 6560 DTIMS-Q-TOF spectrometer, with the dual-ESI source operated in positive ion mode.

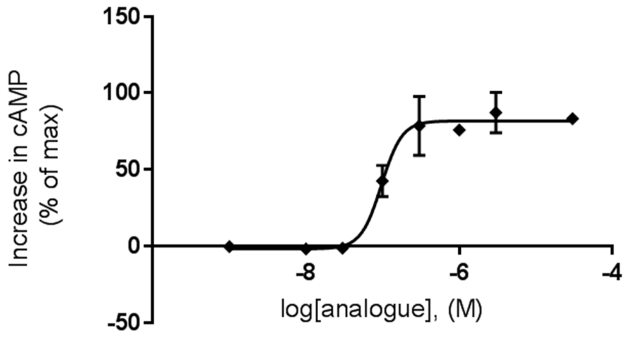

Supplementary Figure 24. In vitro pharmacology characterization. Concentration-response curve for EC_{50} determination of oligomer **4**. Evaluation of the agonist activity at the mouse GLP-1 receptor endogenously expressed in βTC6 cells, was determined by measuring their effects on cAMP production using HTRF detection method. The data are mean ±SEM of a typical experiment performed twice. Source data are provided as a Source Data file (Table 1, Fig. 2 tab).

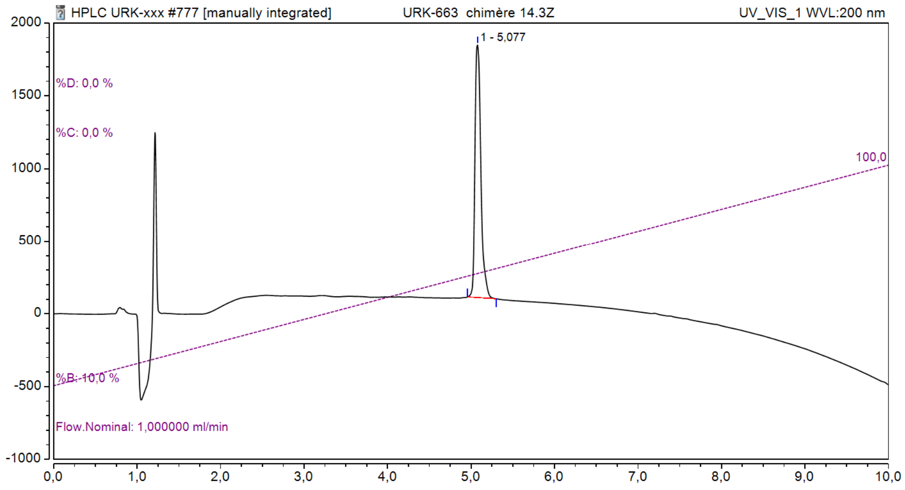

Supplementary Figure 25. HPLC profile. Oligomer **5** (10-100%; CH_3CN 0.1 % TFA in H_2O 0.1% TFA, 10 min, C18)

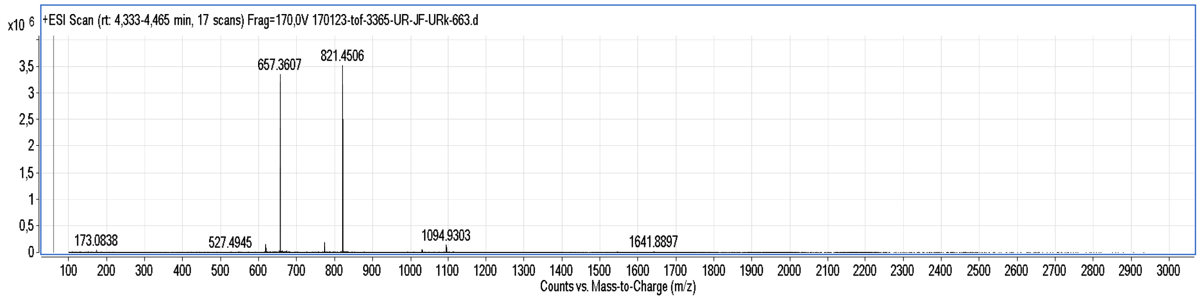

Supplementary Figure 26. LC-MS spectrum of Oligomer 5. Characterization of oligomer **5** by LC-MS. The experiment was performed on UHPLC (Agilent 1290 Infinity) coupled to an ESI -MS Tof (Agilent 6230 ESI) with the dual-ESI source operated in positive ion mode. (Gradient: 10-100%; CH_3CN 0.1 % Formic acid in H_2O 0.1% formic acid, 6 min, C18).

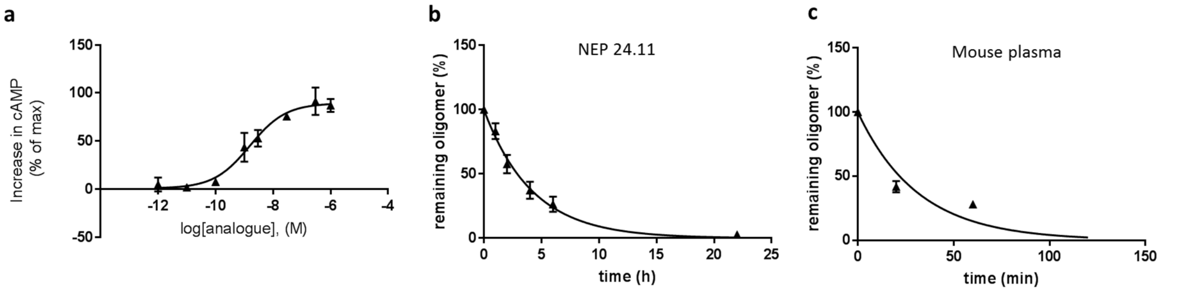

Supplementary Figure 27. In vitro pharmacology characterization. (a) Concentration-response curve for EC_{50} determination of oligomer **5**. Evaluation of the agonist activity at the mouse GLP-1 receptor endogenously expressed in βTC6 cells, was determined by measuring their effects on cAMP production using HTRF detection method. The data are mean ±SEM of a typical experiment performed twice. (b) Enzymatic degradation (NEP 24.11). The data are mean ±SEM of a typical experiment performed 3 times. (c) Mouse plasma degradation. The data are mean ±SEM of a typical experiment performed 3 times. Source data are provided as a Source Data file ((a): Table 1, Fig. 2 tab. (b-c): Table 2 tab).

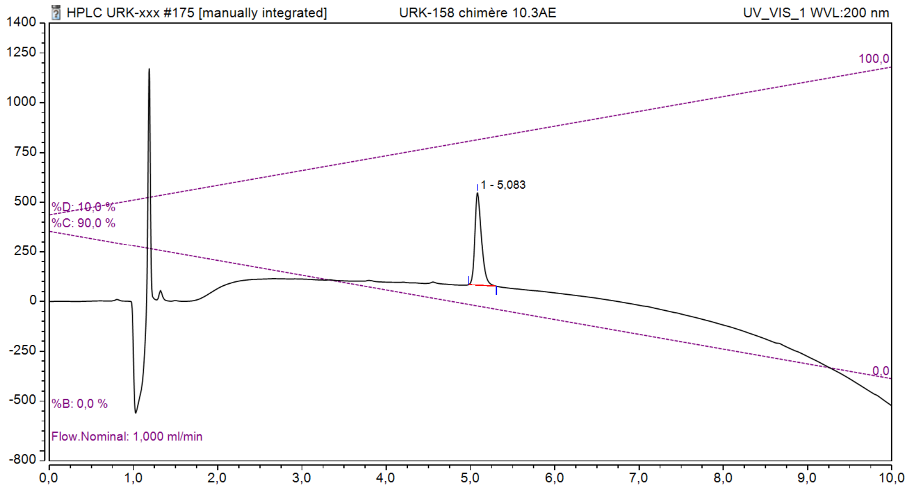

Supplementary Figure 28. HPLC profile. Oligomer **6** (10-100%; CH_3CN 0.1 % TFA in H_2O 0.1% TFA, 10 min, C18)

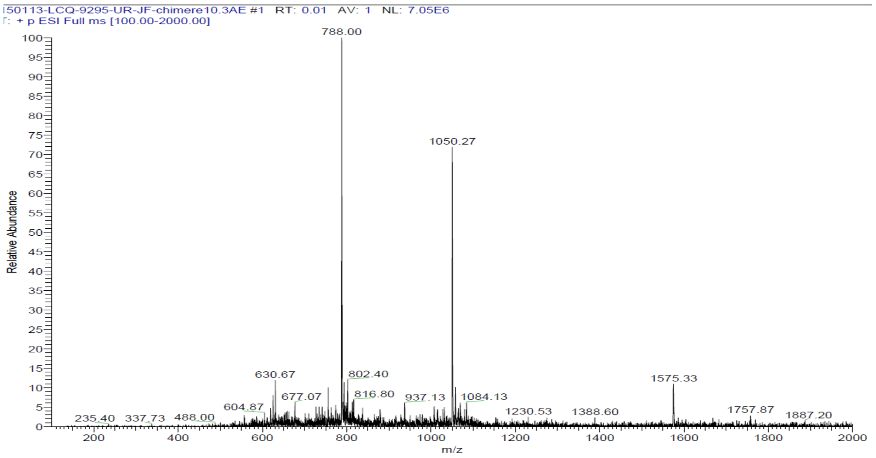

Supplementary Figure 29. Electrospray ionization mass spectrum (ESI-MS) of oligomer 6. The experiments was performed on an Agilent 6560 DTIMS-Q-TOF spectrometer, with the dual-ESI source operated in positive ion mode.

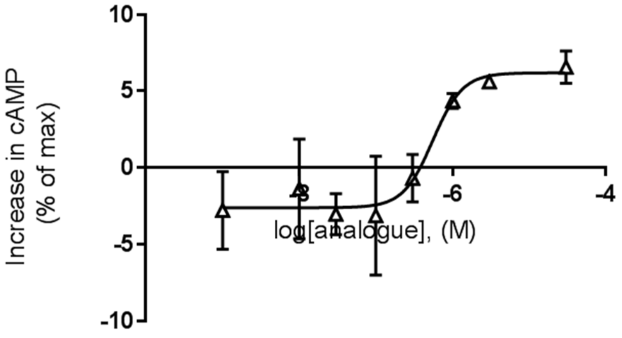

Supplementary Figure 30. In vitro pharmacology characterization. Concentration-response curve for EC_{50} determination of oligomer **6**. Evaluation of the agonist activity at the mouse GLP-1 receptor endogenously expressed in βTC6 cells, was determined by measuring their effects on cAMP production using HTRF detection method. The data are mean ±SEM of a typical experiment performed twice. Source data are provided as a Source Data file (Table 1, Fig. 2 tab).

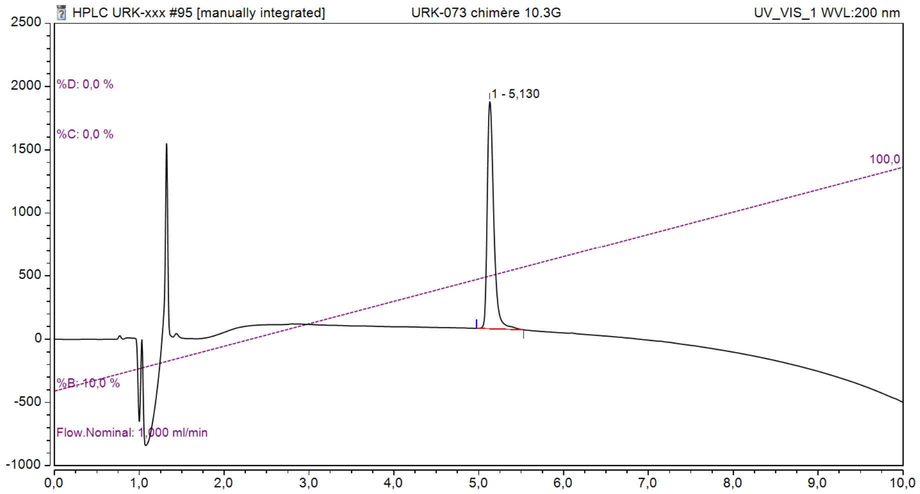

Supplementary Figure 31. HPLC profile. Oligomer **7** (10-100%; CH_3CN 0.1 % TFA in H_2O 0.1% TFA, 10 min, C18)

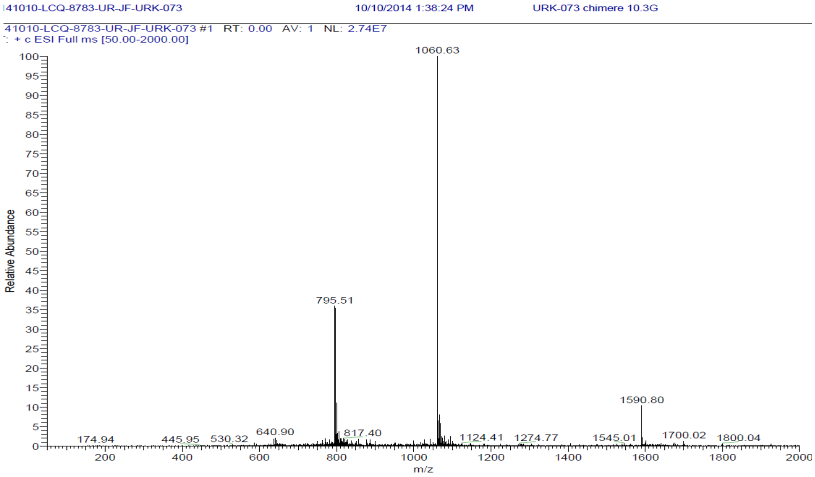

Supplementary Figure 32. Electrospray ionization mass spectrum (ESI-MS) of oligomer 7. The experiment was performed on an Agilent 6560 DTIMS-Q-TOF spectrometer, with the dual-ESI source operated in positive ion mode.

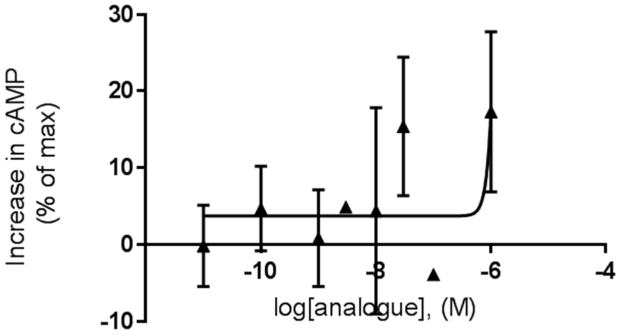

Supplementary Figure 33. In vitro pharmacology characterization. Concentration-response curve for EC_{50} determination of oligomer **7**. Evaluation of the agonist activity at the mouse GLP-1 receptor endogenously expressed in βTC6 cells, was determined by measuring their effects on cAMP production using HTRF detection method. The data are mean ±SEM of a typical experiment performed twice. Source data are provided as a Source Data file (Table 1, Fig. 2 tab).

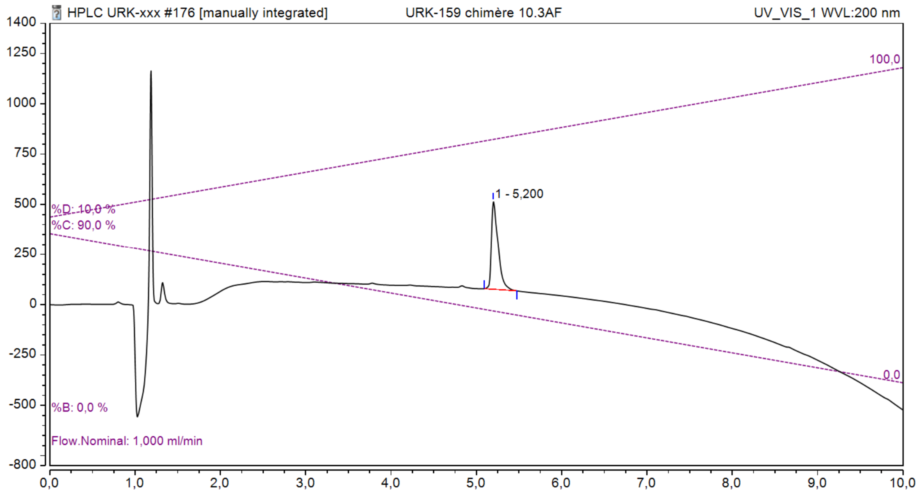

Supplementary Figure 34. HPLC profile. Oligomer **8** (10-100%; CH_3CN 0.1 % TFA in H_2O 0.1% TFA, 10 min, C18)

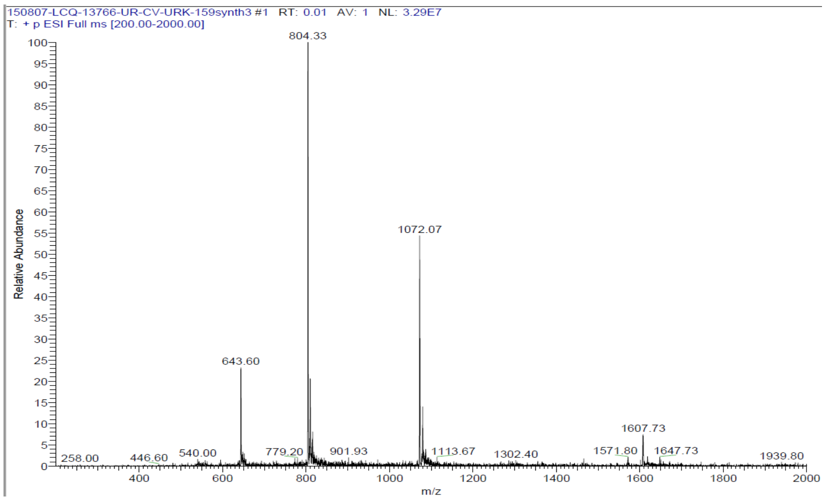

Supplementary Figure 35. Electrospray ionization mass spectrum (ESI-MS) of oligomer 8. The experiment was performed on an Agilent 6560 DTIMS-Q-TOF spectrometer, with the dual-ESI source operated in positive ion mode.

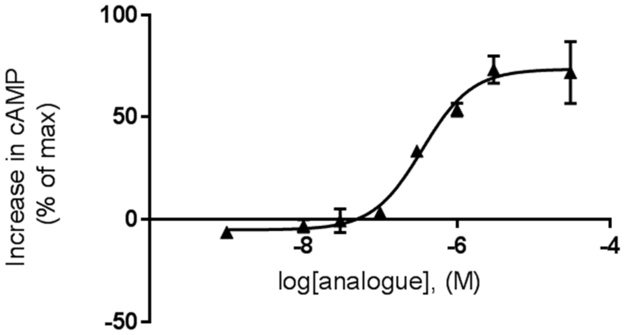

Supplementary Figure 36. In vitro pharmacology characterization. Concentration-response curve for EC_{50} determination of oligomer **8**. Evaluation of the agonist activity at the mouse GLP-1 receptor endogenously expressed in βTC6 cells, was determined by measuring their effects on cAMP production using HTRF detection method. The data are mean ±SEM of a typical experiment performed twice. Source data are provided as a Source Data file (Table 1, Fig. 2 tab).

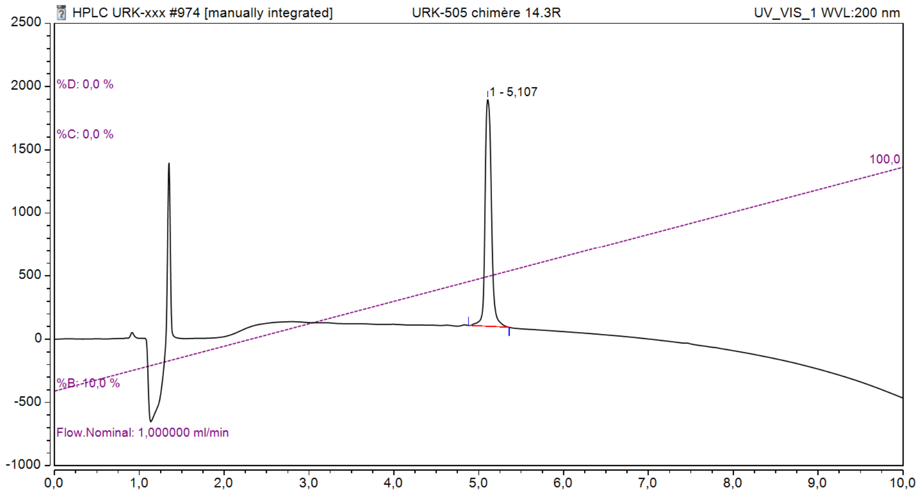

Supplementary Figure 37. HPLC profile. Oligomer **9** (10-100%; CH_3CN 0.1 % TFA in H_2O 0.1% TFA, 10 min, C18)

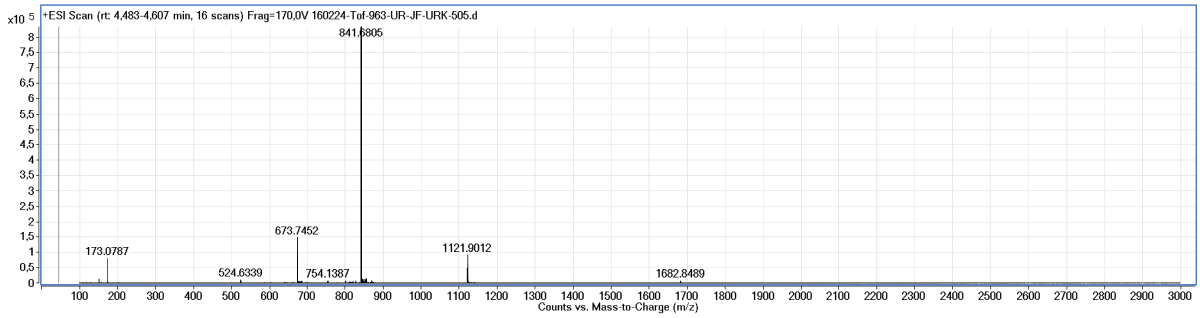

Supplementary Figure 38. LC-MS spectrum of Oligomer 9. Characterization of oligomer **9** by LC-MS. The experiment was performed on UHPLC (Agilent 1290 Infinity) coupled to an ESI -MS Tof (Agilent 6230 ESI) with the dual-ESI source operated in positive ion mode. (Gradient: 10-100%; CH_3CN 0.1 % Formic acid in H_2O 0.1% formic acid, 6 min, C18).

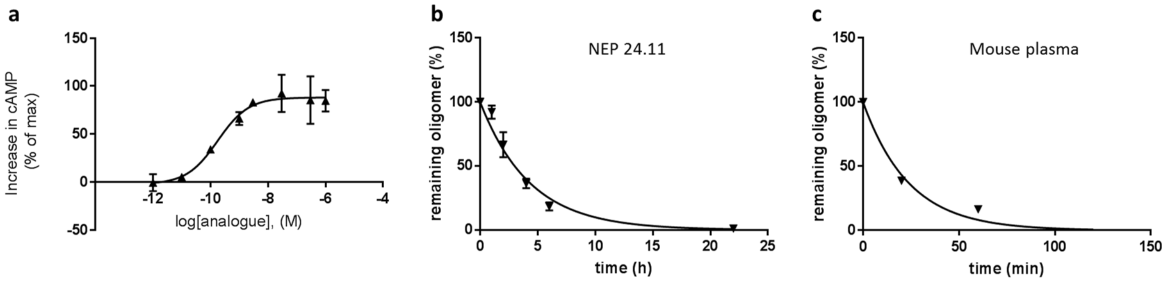

Supplementary Figure 39. In vitro pharmacology characterization. (a) Concentration-response curve for EC_{50} determination of oligomer **9**. Evaluation of the agonist activity at the mouse GLP-1 receptor endogenously expressed in βTC6 cells, was determined by measuring their effects on cAMP production using HTRF detection method. The data are mean ±SEM of a typical experiment performed twice. (b) Enzymatic degradation (NEP 24.11). The data are mean ±SEM of a typical experiment performed 3 times. (c) Mouse plasma degradation. The data are mean ±SEM of a typical experiment performed 3 times. Source data are provided as a Source Data file ((a): Table 1, Fig. 2 tab. (b-c): Table 2 tab).

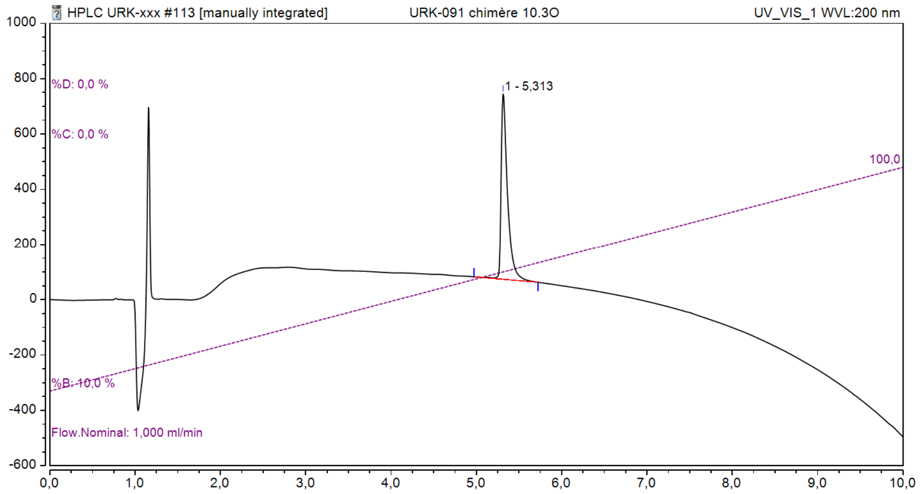

Supplementary Figure 40. HPLC profile. Oligomer **10** (10-100%; CH_3CN 0.1 % TFA in H_2O 0.1% TFA, 10 min, C18)

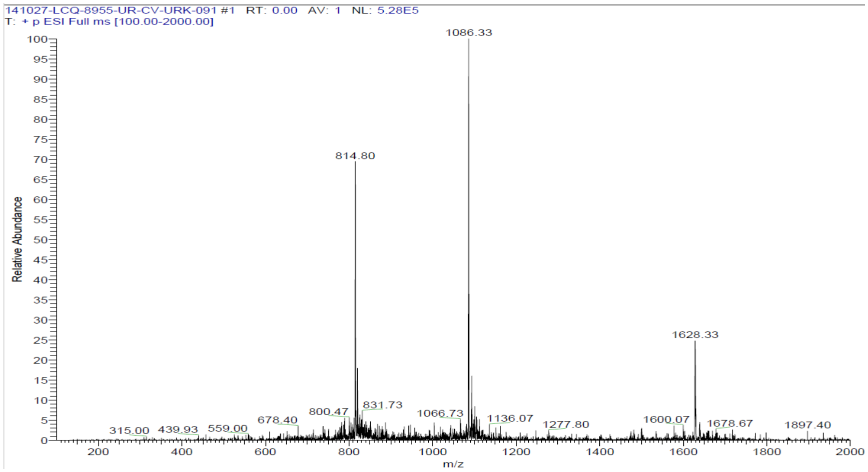

Supplementary Figure 41. Electrospray ionization mass spectrum (ESI-MS) of oligomer 10. The experiment was performed on an Agilent 6560 DTIMS-Q-TOF spectrometer, with the dual-ESI source operated in positive ion mode.

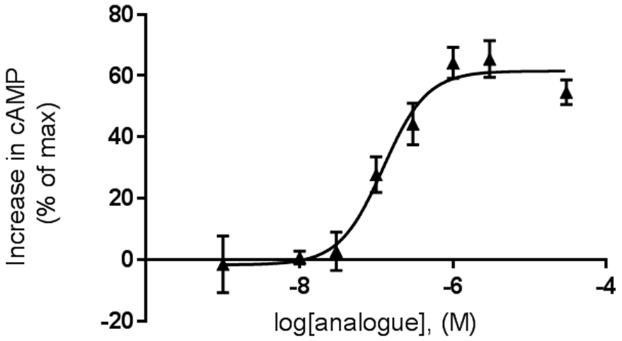

Supplementary Figure 42. In vitro pharmacology characterization. Concentration-response curve for EC_{50} determination of oligomer **10**. Evaluation of the agonist activity at the mouse GLP-1 receptor endogenously expressed in βTC6 cells, was determined by measuring their effects on cAMP production using HTRF detection method. The data are mean ±SEM of a typical experiment performed twice. Source data are provided as a Source Data file (Table 1, Fig. 2 tab).

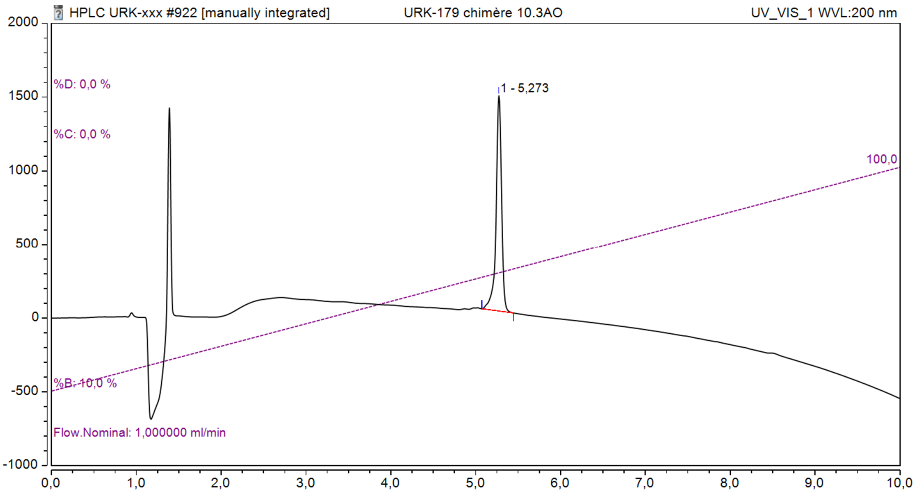

Supplementary Figure 43. HPLC profile. Oligomer **11** (10-100%; CH_3CN 0.1 % TFA in H_2O 0.1% TFA, 10 min, C18)

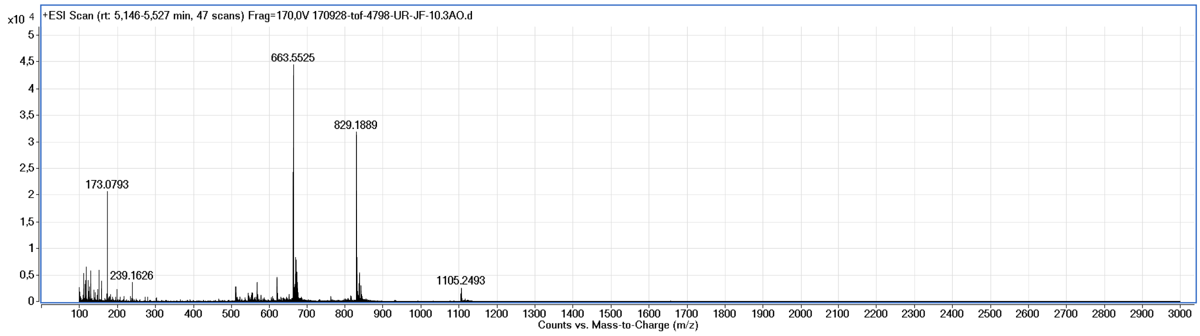

Supplementary Figure 44. LC-MS spectrum of Oligomer 11. Characterization of oligomer **11** by LC-MS. The experiment was performed on UHPLC (Agilent 1290 Infinity) coupled to an ESI -MS Tof (Agilent 6230 ESI) with the dual-ESI source operated in positive ion mode. (Gradient: 10-100%; CH_3CN 0.1 % Formic acid in H_2O 0.1% formic acid, 6 min, C18).

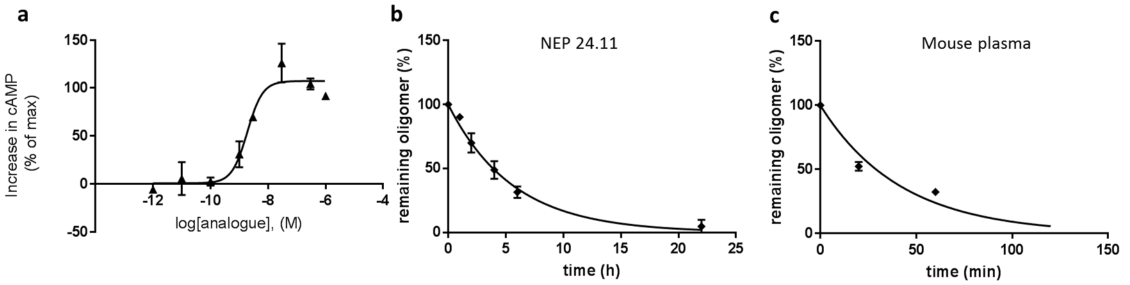

Supplementary Figure 45. In vitro pharmacology characterization. (a) Concentration-response curve for EC_{50} determination of oligomer **11**. Evaluation of the agonist activity at the mouse GLP-1 receptor endogenously expressed in βTC6 cells, was determined by measuring their effects on cAMP production using HTRF detection method. The data are mean ±SEM of a typical experiment performed twice. (b) Enzymatic degradation (NEP 24.11). The data are mean ±SEM of a typical experiment performed 3 times. (c) Mouse plasma degradation. The data are mean ±SEM of a typical experiment performed 3 times. Source data are provided as a Source Data file ((a): Table 1, Fig. 2 tab. (b-c): Table 2 tab).

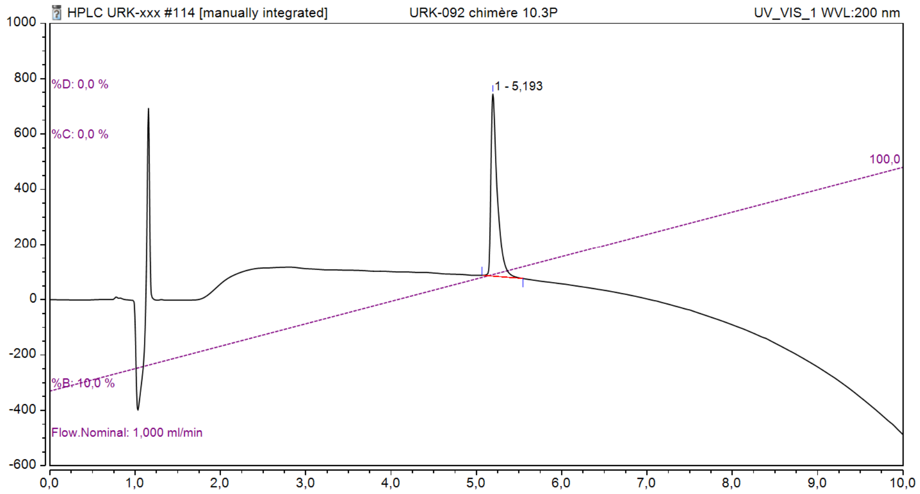

Supplementary Figure 46. HPLC profile. Oligomer **12** (10-100%; CH_3CN 0.1 % TFA in H_2O 0.1% TFA, 10 min, C18)

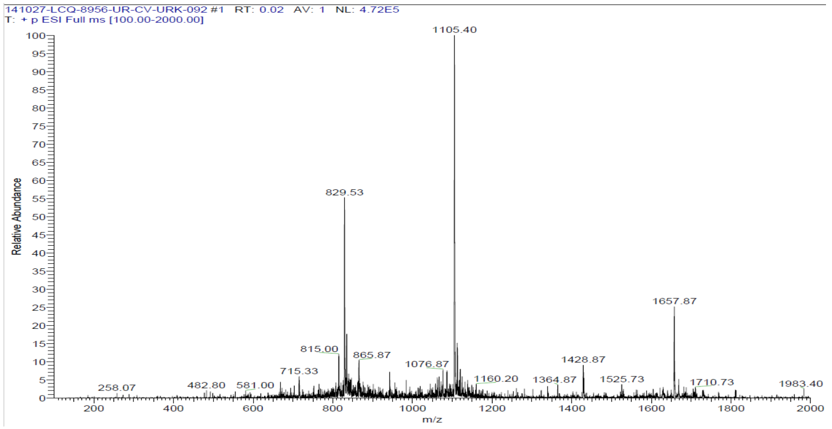

Supplementary Figure 47. Electrospray ionization mass spectrum (ESI-MS) of oligomer 12. The experiment was performed on an Agilent 6560 DTIMS-Q-TOF spectrometer, with the dual-ESI source operated in positive ion mode.

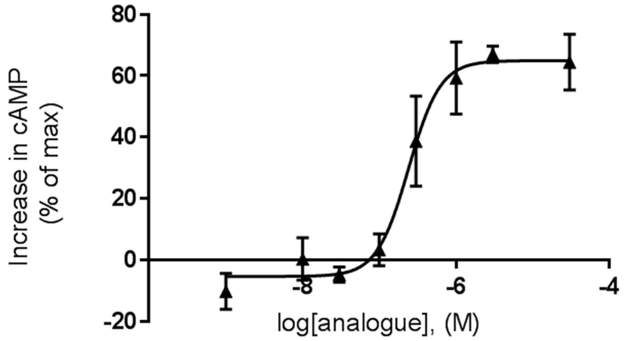

Supplementary Figure 48. In vitro pharmacology characterization. Concentration-response curve for EC_{50} determination of oligomer **12**. Evaluation of the agonist activity at the mouse GLP-1 receptor endogenously expressed in βTC6 cells, was determined by measuring their effects on cAMP production using HTRF detection method. The data are mean ±SEM of a typical experiment performed twice. Source data are provided as a Source Data file (Table 1, Fig. 2 tab).

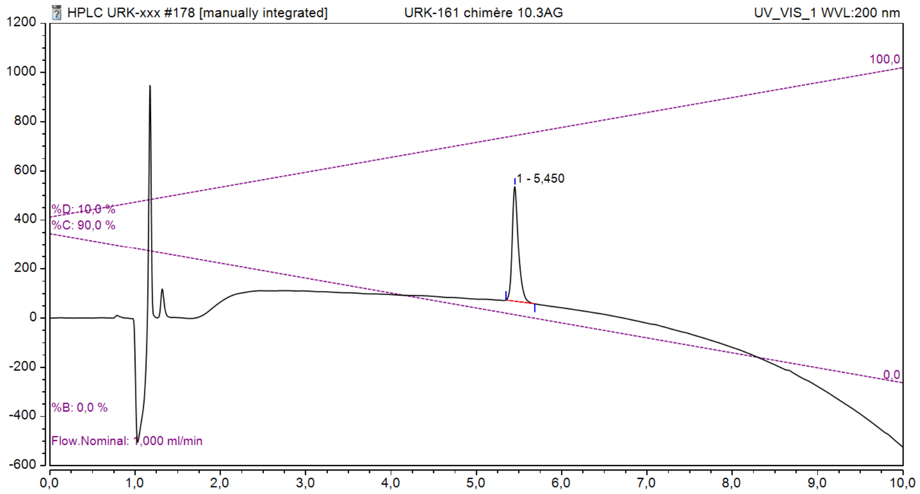

Supplementary Figure 49. HPLC profile. Oligomer **13** (10-100%; CH_3CN 0.1 % TFA in H_2O 0.1% TFA, 10 min, C18)

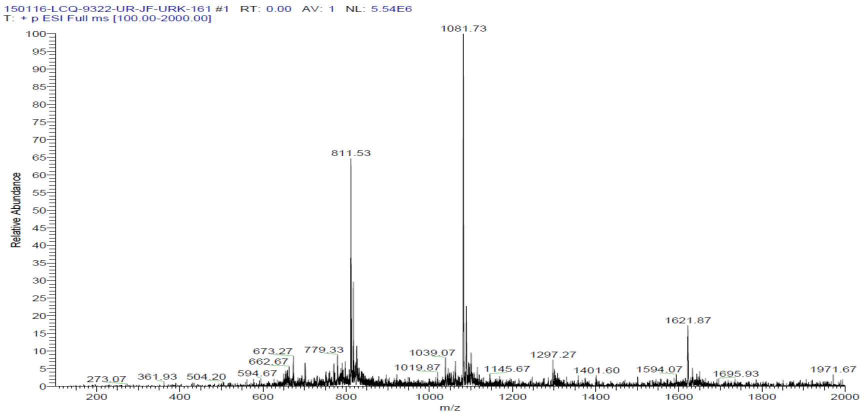

Supplementary Figure 50. Electrospray ionization mass spectrum (ESI-MS) of oligomer 13. The experiment was performed on an Agilent 6560 DTIMS-Q-TOF spectrometer, with the dual-ESI source operated in positive ion mode.

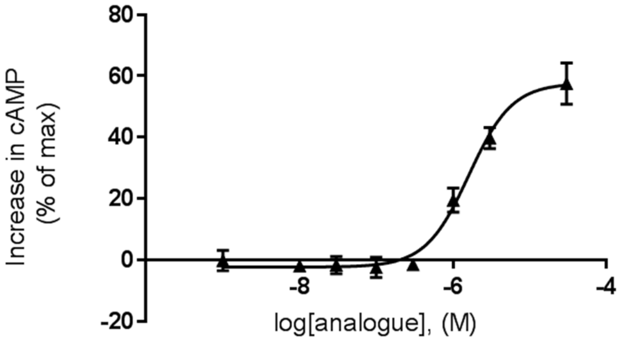

Supplementary Figure 51. In vitro pharmacology characterization. Concentration-response curve for EC_{50} determination of oligomer **13**. Evaluation of the agonist activity at the mouse GLP-1 receptor endogenously expressed in βTC6 cells, was determined by measuring their effects on cAMP production using HTRF detection method. The data are mean ±SEM of a typical experiment performed twice. Source data are provided as a Source Data file (Table 1, Fig. 2 tab).

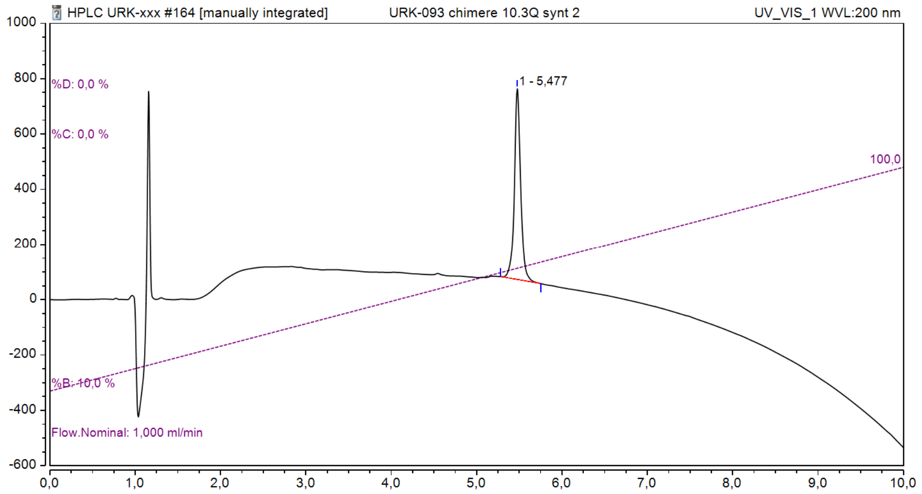

Supplementary Figure 52. HPLC profile. Oligomer **14** (10-100%; CH_3CN 0.1 % TFA in H_2O 0.1% TFA, 10 min, C18)

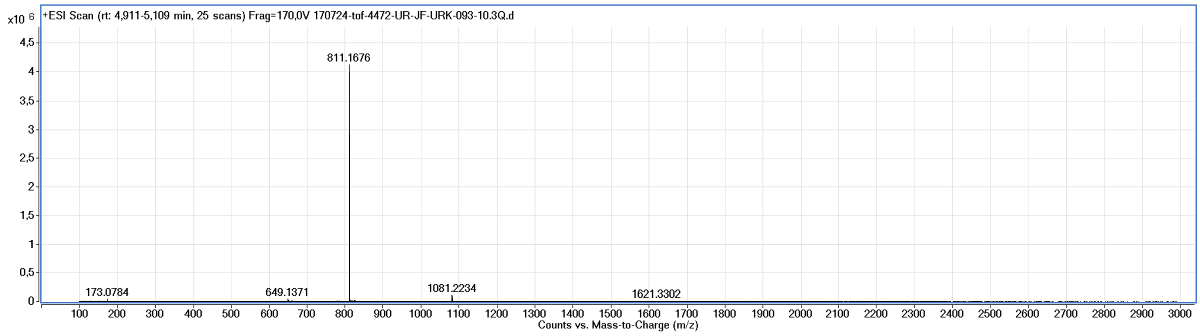

Supplementary Figure 53. LC-MS spectrum of Oligomer 14. Characterization of oligomer **14** by LC-MS. The experiment was performed on UHPLC (Agilent 1290 Infinity) coupled to an ESI -MS Tof (Agilent 6230 ESI) with the dual-ESI source operated in positive ion mode. (Gradient: 10-100%; CH_3CN 0.1 % Formic acid in H_2O 0.1% formic acid, 6 min, C18).

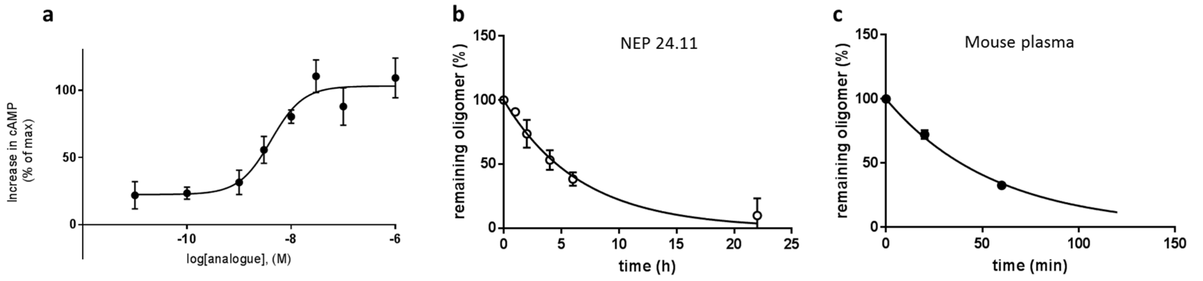

Supplementary Figure 54. In vitro pharmacology characterization. (a) Concentration-response curve for EC_{50} determination of oligomer **14**. Evaluation of the agonist activity at the mouse GLP-1 receptor endogenously expressed in βTC6 cells, was determined by measuring their effects on cAMP production using HTRF detection method. The data are mean ±SEM of a typical experiment performed twice. (b) Enzymatic degradation (NEP 24.11). The data are mean ±SEM of a typical experiment performed 3 times. (c) Mouse plasma degradation. The data are mean ±SEM of a typical experiment performed 3 times. Source data are provided as a Source Data file ((a): Table 1, Fig. 2 tab. (b-c): Table 2 tab).

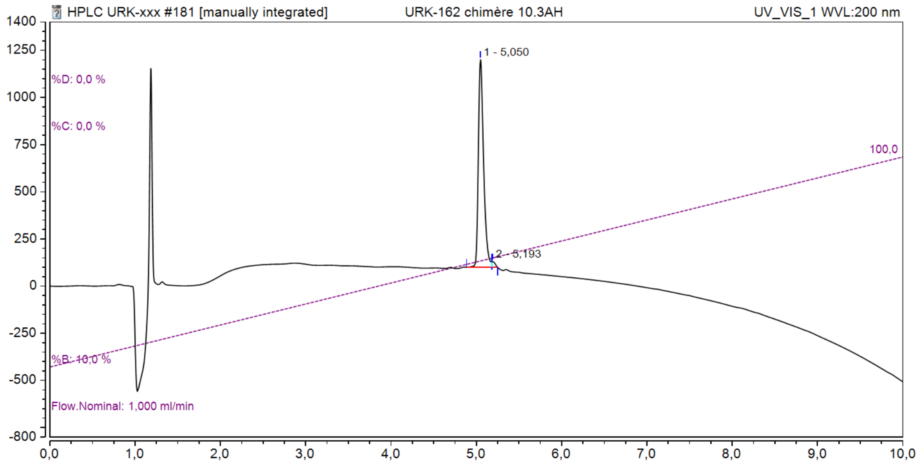

Supplementary Figure 55. HPLC profile. Oligomer **15** (10-100%; CH_3CN 0.1 % TFA in H_2O 0.1% TFA, 10 min, C18)

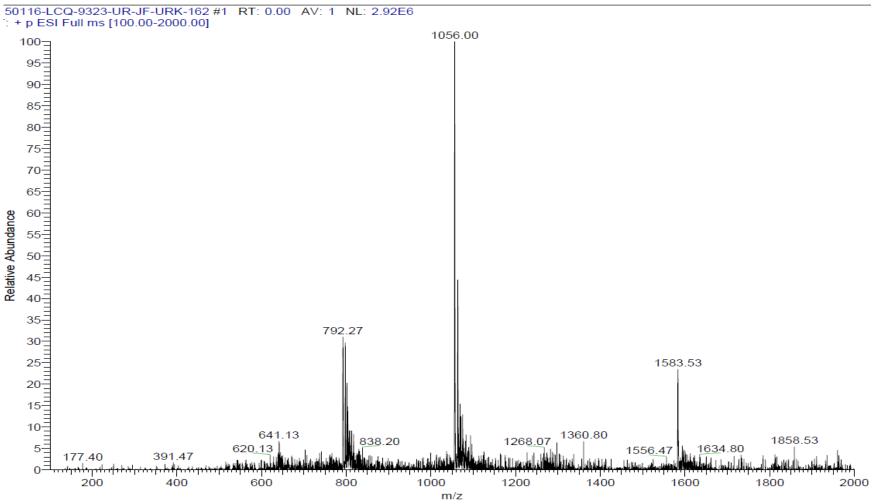

Supplementary Figure 56. Electrospray ionization mass spectrum (ESI-MS) of oligomer 15. The experiment was performed on an Agilent 6560 DTIMS-Q-TOF spectrometer, with the dual-ESI source operated in positive ion mode.

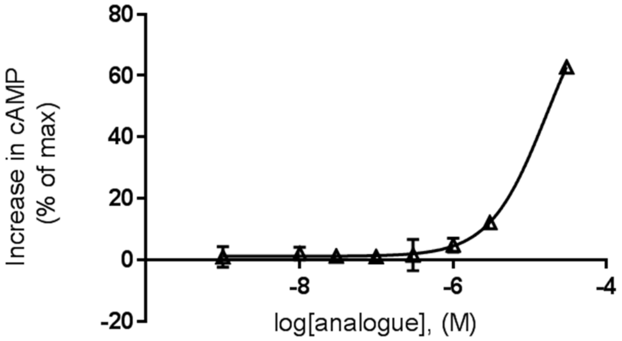

Supplementary Figure 57. In vitro pharmacology characterization. Concentration-response curve for EC_{50} determination of oligomer **15**. Evaluation of the agonist activity at the mouse GLP-1 receptor endogenously expressed in βTC6 cells, was determined by measuring their effects on cAMP production using HTRF detection method. The data are mean ±SEM of a typical experiment performed twice. Source Data are provided as a Source Data file (Table 1, Fig. 2 tab).

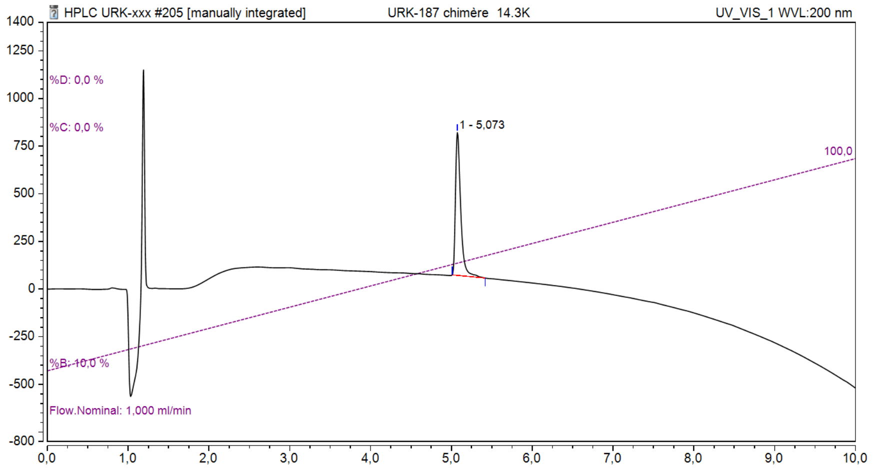

Supplementary Figure 58. HPLC profile. Oligomer **16** (10-100%; CH_3CN 0.1 % TFA in H_2O 0.1% TFA, 10 min, C18)

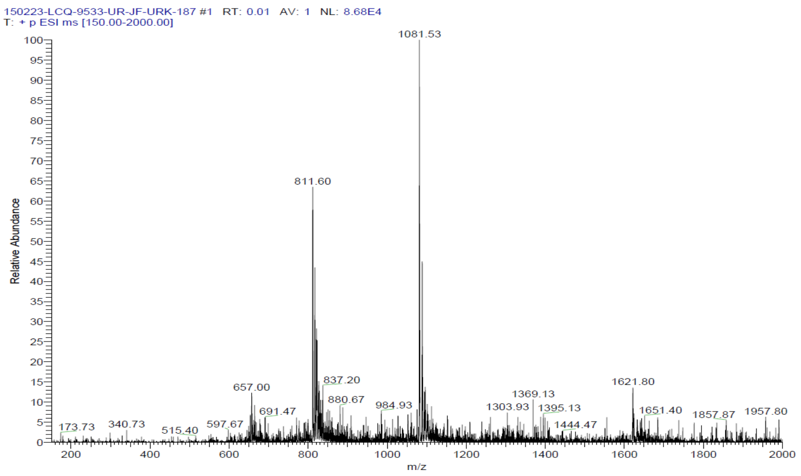

Supplementary Figure 59. Electrospray ionization mass spectrum (ESI-MS) of oligomer 16. The experiment was performed on an Agilent 6560 DTIMS-Q-TOF spectrometer, with the dual-ESI source operated in positive ion mode.

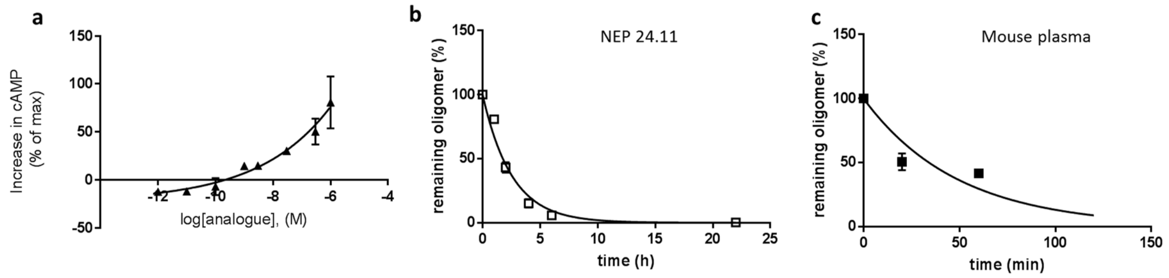

Supplementary Figure 60. In vitro pharmacology characterization. (a) Concentration-response curve for EC_{50} determination of oligomer **16**. Evaluation of the agonist activity at the mouse GLP-1 receptor endogenously expressed in βTC6 cells, was determined by measuring their effects on cAMP production using HTRF detection method. The data are mean ±SEM of a typical experiment performed twice. (b) Enzymatic degradation (NEP 24.11). The data are mean ±SEM of a typical experiment performed 3 times. (c) Mouse plasma degradation. The data are mean ±SEM of a typical experiment performed 3 times. Source data are provided as a Source Data file ((a): Table 1, Fig. 2 tab. (b-c): Table 2 tab).

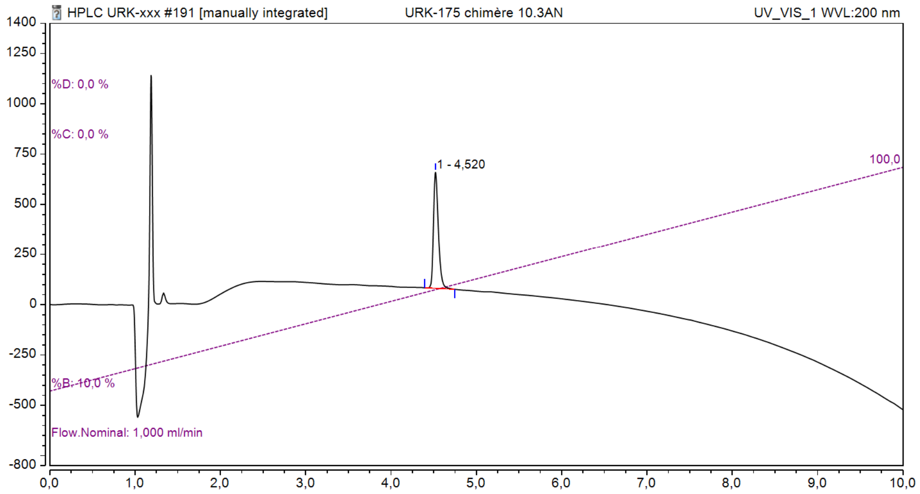

Supplementary Figure 61. HPLC profile. Oligomer **17** (10-100%; CH_3CN 0.1 % TFA in H_2O 0.1% TFA, 10 min, C18)

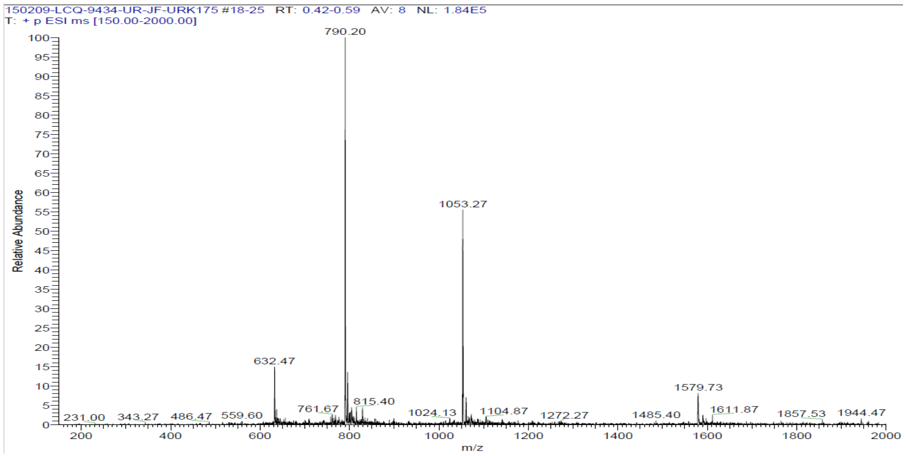

Supplementary Figure 62. Electrospray ionization mass spectrum (ESI-MS) of oligomer 17. The experiment was performed on an Agilent 6560 DTIMS-Q-TOF spectrometer, with the dual-ESI source operated in positive ion mode.

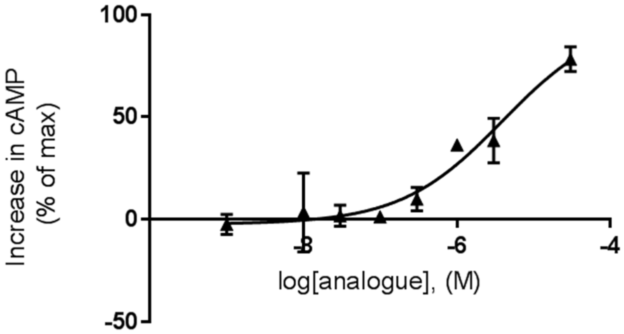

Supplementary Figure 63. In vitro pharmacology characterization. Concentration-response curve for EC_{50} determination of oligomer 17. Evaluation of the agonist activity at the mouse GLP-1 receptor endogenously expressed in βTC6 cells, was determined by measuring their effects on cAMP production using HTRF detection method. The data are mean ±SEM of a typical experiment performed twice. Source data are provided as a Source Data file (Table 1, Fig. 2 tab).

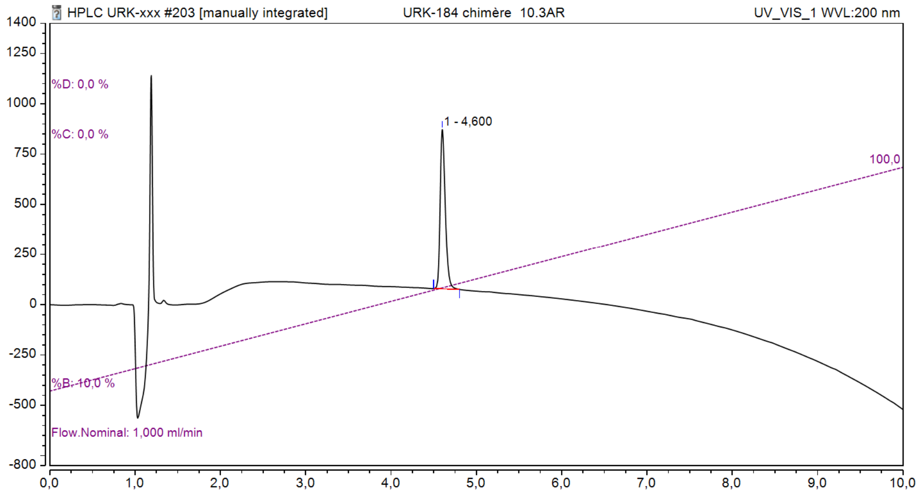

Supplementary Figure 64. HPLC profile. Oligomer **18** (10-100%; CH_3CN 0.1 % TFA in H_2O 0.1% TFA, 10 min, C18)

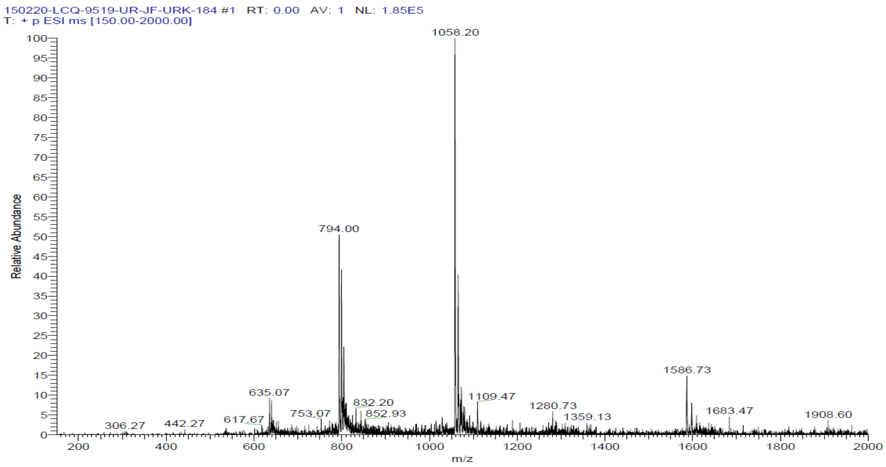

Supplementary Figure 65. Electrospray ionization mass spectrum (ESI-MS) of oligomer 18. The experiment was performed on an Agilent 6560 DTIMS-Q-TOF spectrometer, with the dual-ESI source operated in positive ion mode.

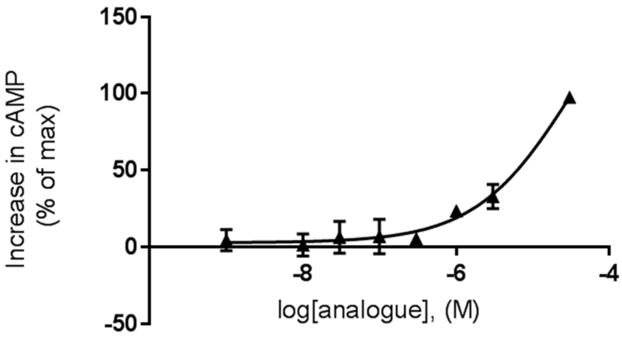

Supplementary Figure 66. In vitro pharmacology characterization. Concentration-response curve for EC_{50} determination of oligomer **18**. Evaluation of the agonist activity at the mouse GLP-1 receptor endogenously expressed in βTC6 cells, was determined by measuring their effects on cAMP production using HTRF detection method. The data are mean ±SEM of a typical experiment performed twice. Source data are provided as a Source Data file (Table 1, Fig. 2 tab).

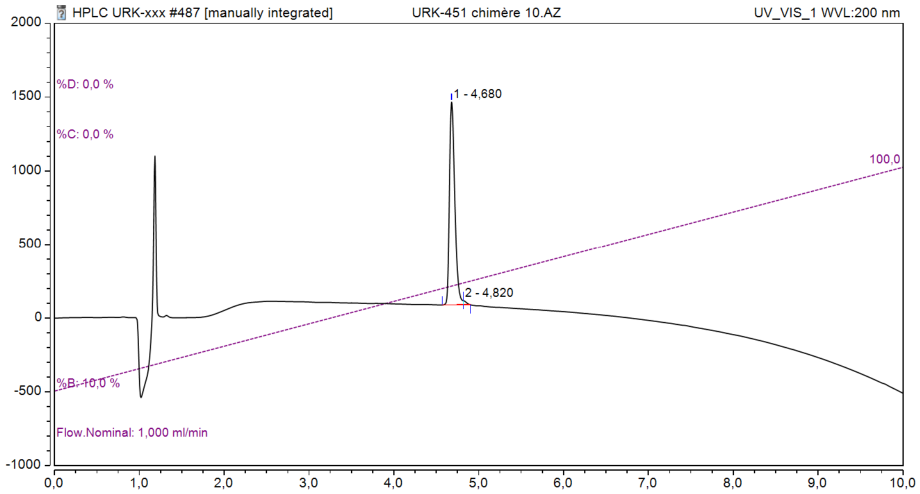

Supplementary Figure 67. HPLC profile. Oligomer **19** (10-100%; CH_3CN 0.1 % TFA in H_2O 0.1% TFA, 10 min, C18)

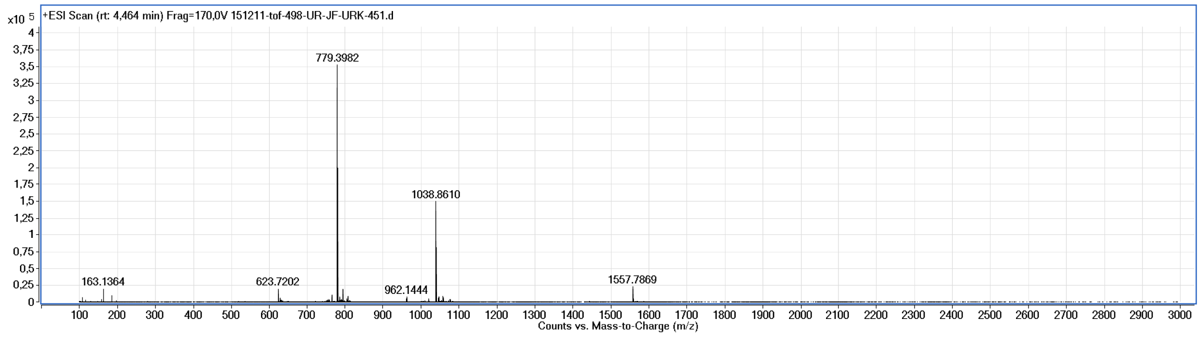

Supplementary Figure 68. LC-MS spectrum of Oligomer 19. Characterization of oligomer **19** by LC-MS. The experiment was performed on UHPLC (Agilent 1290 Infinity) coupled to an ESI -MS Tof (Agilent 6230 ESI) with the dual-ESI source operated in positive ion mode. (Gradient: 10-100%; CH_3CN 0.1 % Formic acid in H_2O 0.1% formic acid, 6 min, C18).

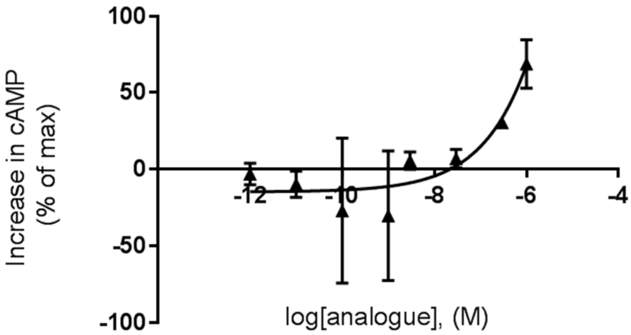

Supplementary Figure 69. In vitro pharmacology characterization. Concentration-response curve for EC_{50} determination of oligomer **19**. Evaluation of the agonist activity at the mouse GLP-1 receptor endogenously expressed in βTC6 cells, was determined by measuring their effects on cAMP production using HTRF detection method. The data are mean ±SEM of a typical experiment performed twice. Source data are provided as a Source Data file (Table 1, Fig. 2 tab).

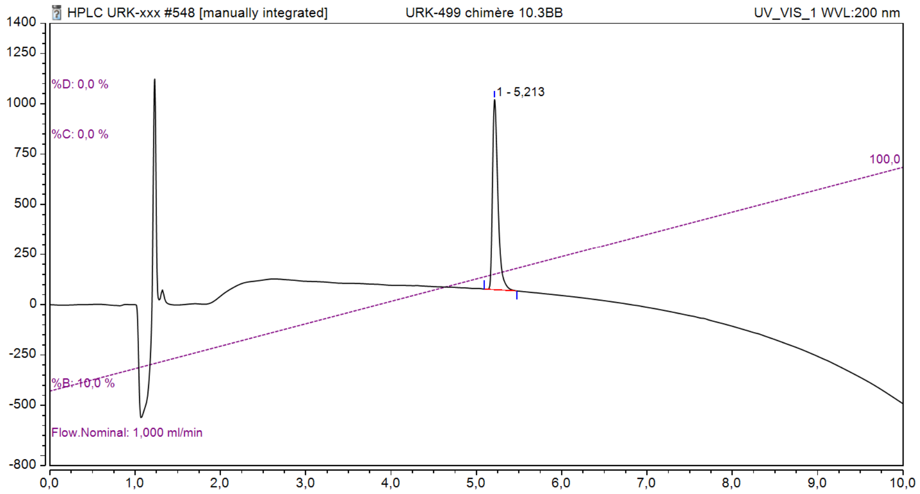

Supplementary Figure 70. HPLC profile. Oligomer **20** (10-100%; CH_3CN 0.1 % TFA in H_2O 0.1% TFA, 10 min, C18)

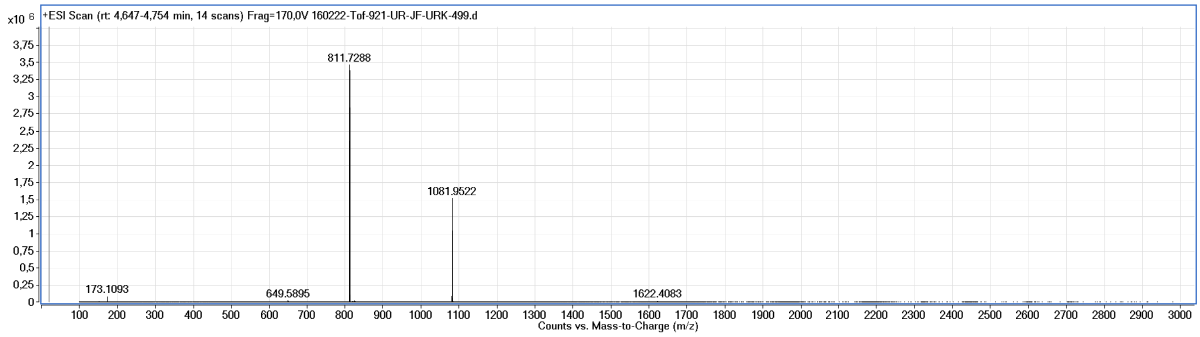

Supplementary Figure 71. LC-MS spectrum of Oligomer 20. Characterization of oligomer **20** by LC-MS. The experiment was performed on UHPLC (Agilent 1290 Infinity) coupled to an ESI -MS Tof (Agilent 6230 ESI) with the dual-ESI source operated in positive ion mode. (Gradient: 10-100%; CH_3CN 0.1 % Formic acid in H_2O 0.1% formic acid, 6 min, C18).

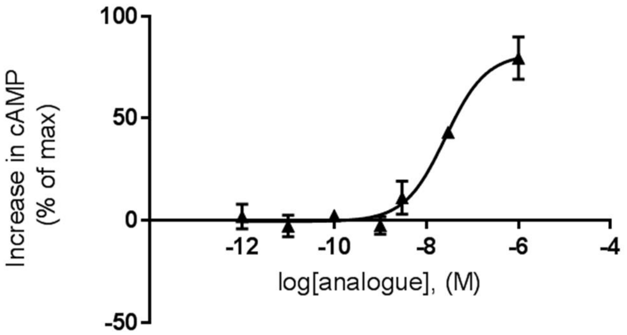

Supplementary Figure 72. In vitro pharmacology characterization. Concentration-response curve for EC_{50} determination of oligomer **20**. Evaluation of the agonist activity at the mouse GLP-1 receptor endogenously expressed in βTC6 cells, was determined by measuring their effects on cAMP production using HTRF detection method. The data are mean ±SEM of a typical experiment performed twice. Source data are provided as a Source Data file (Table 1, Fig. 2 tab).

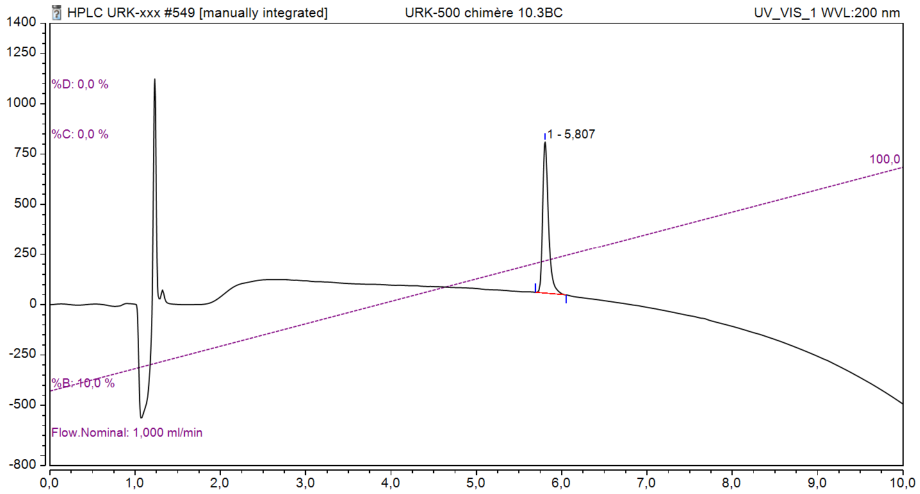

Supplementary Figure 73. HPLC profile. Oligomer **21** (10-100%; CH_3CN 0.1 % TFA in H_2O 0.1% TFA, 10 min, C18)

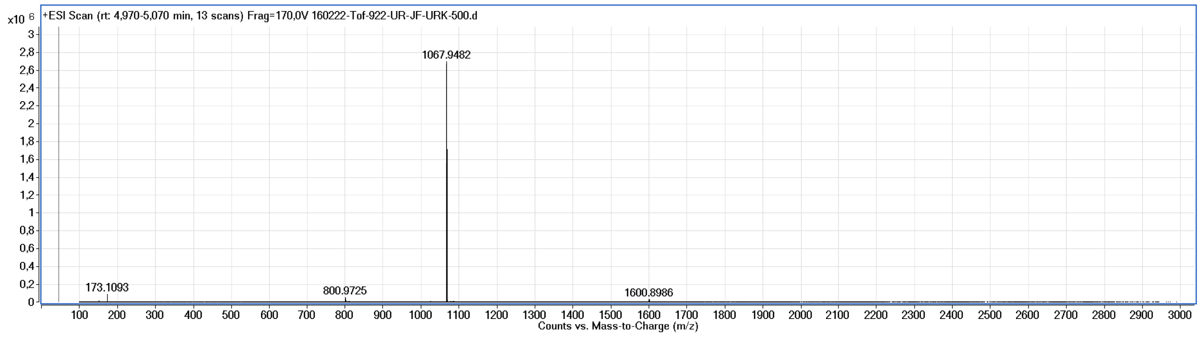

Supplementary Figure 74. LC-MS spectrum of Oligomer 21. Characterization of oligomer **21** by LC-MS. The experiment was performed on UHPLC (Agilent 1290 Infinity) coupled to an ESI -MS Tof (Agilent 6230 ESI) with the dual-ESI source operated in positive ion mode. (Gradient: 10-100%; CH_3CN 0.1 % Formic acid in H_2O 0.1% formic acid, 6 min, C18).

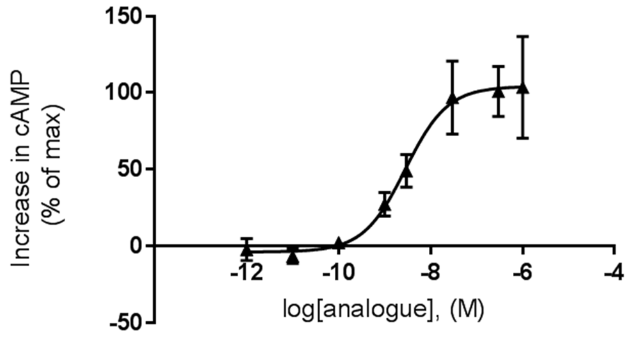

Supplementary Figure 75. In vitro pharmacology characterization. Concentration-response curve for EC_{50} determination of oligomer **21**. Evaluation of the agonist activity at the mouse GLP-1 receptor endogenously expressed in βTC6 cells, was determined by measuring their effects on cAMP production using HTRF detection method. The data are mean ±SEM of a typical experiment performed twice. Source data are provided as a Source Data file (Table 1, Fig. 2 tab).

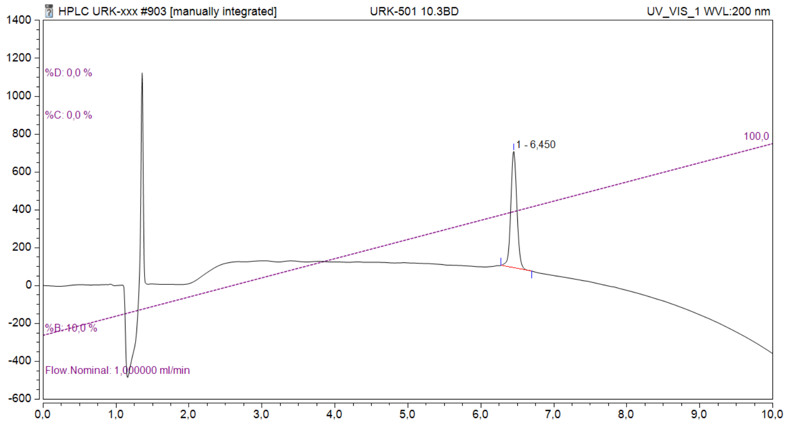

Supplementary Figure 76. HPLC profile. Oligomer **22** (10-100%; CH_3CN 0.1 % TFA in H_2O 0.1% TFA, 10 min, C18)

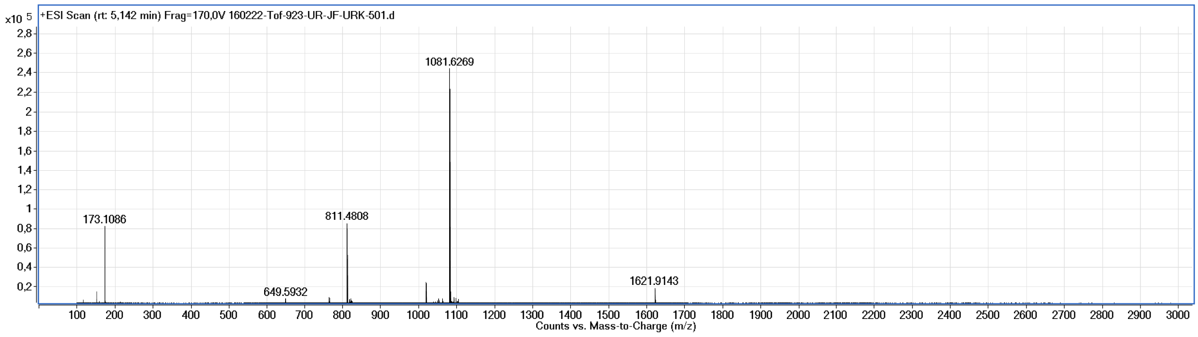

Supplementary Figure 77. LC-MS spectrum of Oligomer 22. Characterization of oligomer **22** by LC-MS. The experiment was performed on UHPLC (Agilent 1290 Infinity) coupled to an ESI -MS Tof (Agilent 6230 ESI) with the dual-ESI source operated in positive ion mode. (Gradient: 10-100%; CH_3CN 0.1 % Formic acid in H_2O 0.1% formic acid, 6 min, C18).

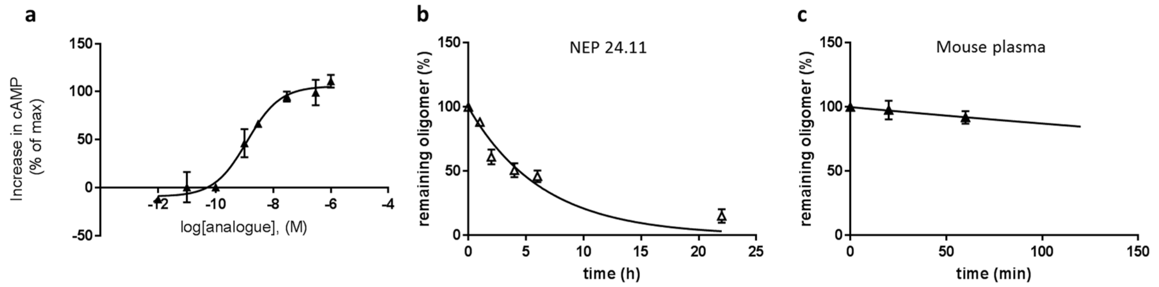

Supplementary Figure 78. In vitro pharmacology characterization. (a) Concentration-response curve for EC_{50} determination of oligomer **22**. Evaluation of the agonist activity at the mouse GLP-1 receptor endogenously expressed in βTC6 cells, was determined by measuring their effects on cAMP production using HTRF detection method. The data are mean ±SEM of a typical experiment performed twice. (b) Enzymatic degradation (NEP 24.11). The data are mean ±SEM of a typical experiment performed 3 times. (c) Mouse plasma degradation. The data are mean ±SEM of a typical experiment performed 3 times. Source data are provided as a Source Data file ((a): Table 1, Fig. 2 tab. (b-c): Table 2 tab)..

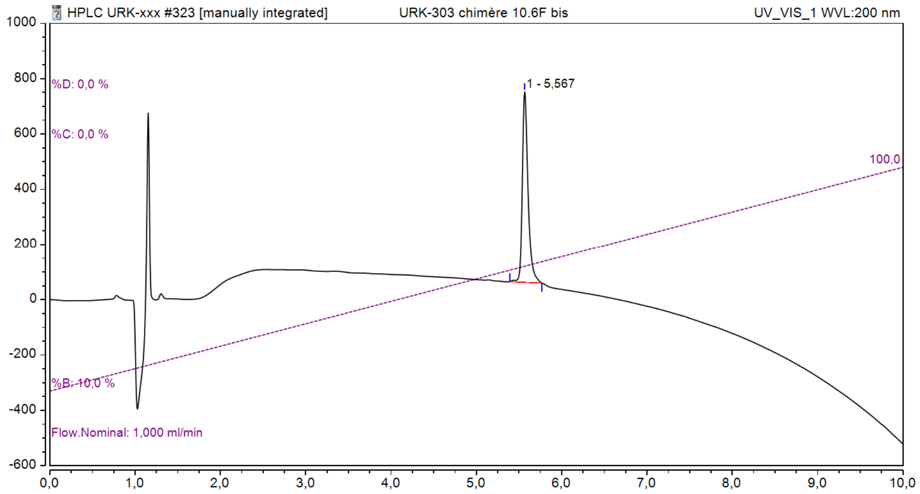

Supplementary Figure 79. HPLC profile. Oligomer **23** (10-100%; CH_3CN 0.1 % TFA in H_2O 0.1% TFA, 10 min, C18)

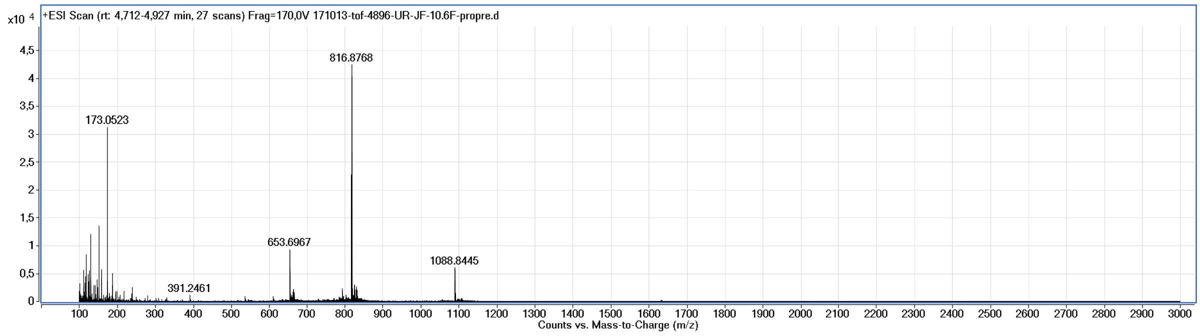

Supplementary Figure 80. LC-MS spectrum of Oligomer 23. Characterization of oligomer **23** by LC-MS. The experiment was performed on UHPLC (Agilent 1290 Infinity) coupled to an ESI -MS Tof (Agilent 6230 ESI) with the dual-ESI source operated in positive ion mode. (Gradient: 10-100%; CH_3CN 0.1 % Formic acid in H_2O 0.1% formic acid, 6 min, C18).

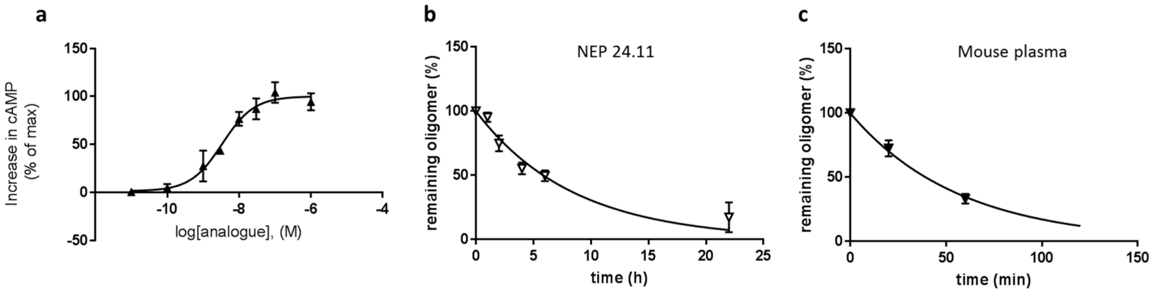

Supplementary Figure 81. In vitro pharmacology characterization. (a) Concentration-response curve for EC_{50} determination of oligomer **23**. Evaluation of the agonist activity at the mouse GLP-1 receptor endogenously expressed in βTC6 cells, was determined by measuring their effects on cAMP production using HTRF detection method. The data are mean ±SEM of a typical experiment performed twice. (b) Enzymatic degradation (NEP 24.11). The data are mean ±SEM of a typical experiment performed 3 times. (c) Mouse plasma degradation. The data are mean ±SEM of a typical experiment performed 3 times. Source data are provided as a Source Data file ((a): Table 1, Fig. 2 tab. (b-c): Table 2 tab).

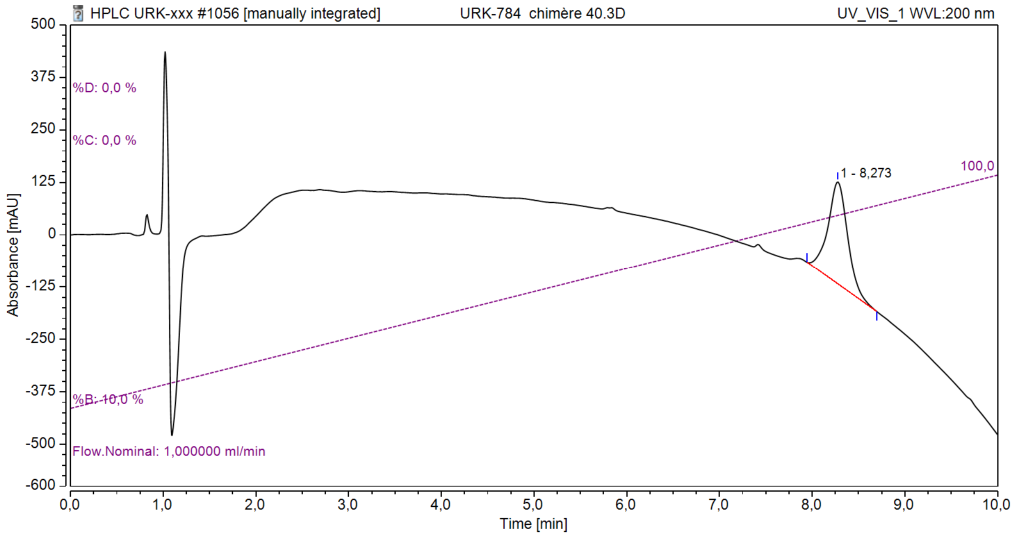

Supplementary Figure 82. HPLC profile. Oligomer **24** (10-100%; CH_3CN 0.1 % TFA in H_2O 0.1% TFA, 10 min, C18)

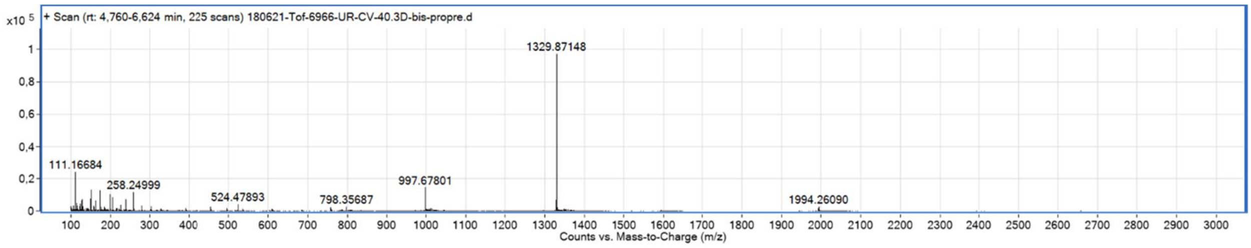

Supplementary Figure 83. LC-MS spectrum of Oligomer 24. Characterization of oligomer **24** by LC-MS. The experiment was performed on UHPLC (Agilent 1290 Infinity) coupled to an ESI -MS Tof (Agilent 6230 ESI) with the dual-ESI source operated in positive ion mode. (Gradient: 10-100%; CH_3CN 0.1 % Formic acid in H_2O 0.1% formic acid, 6 min, C18).

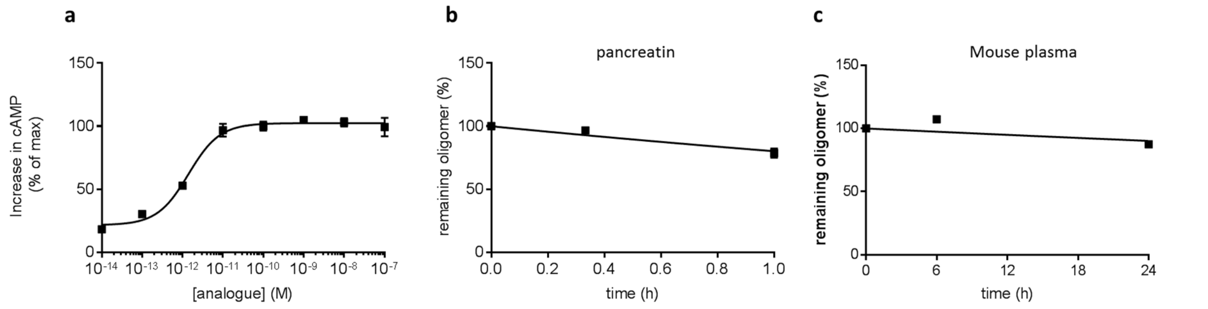

Supplementary Figure 84. In vitro pharmacology characterization. (a) Concentration-response curve (for EC50 determination of oligomer **24.** Evaluation of the agonist activity at the human GLP-1 receptor exogenously expressed in HEK293T cells, was determined by measuring their effects on cAMP production using the HTRF detection method. The data are mean ±SEM of a typical experiment performed 4 times. (b) Enzymatic degradation (Pancreatin). The data are mean ±SEM of a typical experiment performed 3 times. (c) Mouse plasma degradation. The data are mean ±SEM of a typical experiment performed 3 times. Source data are provided as a Source Data file ((a): Supp Fig. 5 tab, (b): Fig. 6b tab, (c): Supp Fig. 3 tab).

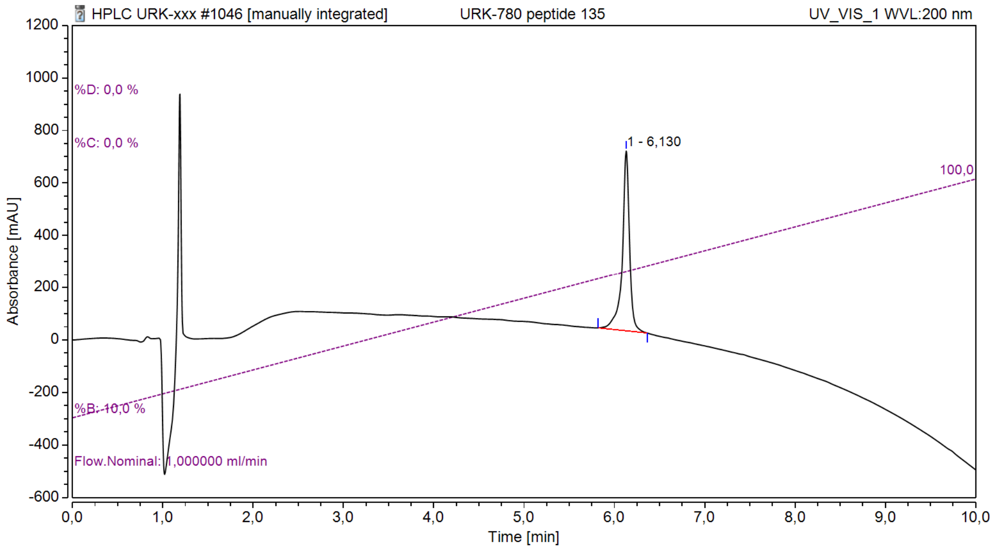

Supplementary Figure 85. HPLC profile. Peptide **25** (10-100%; CH_3CN 0.1 % TFA in H_2O 0.1% TFA, 10 min, C18)

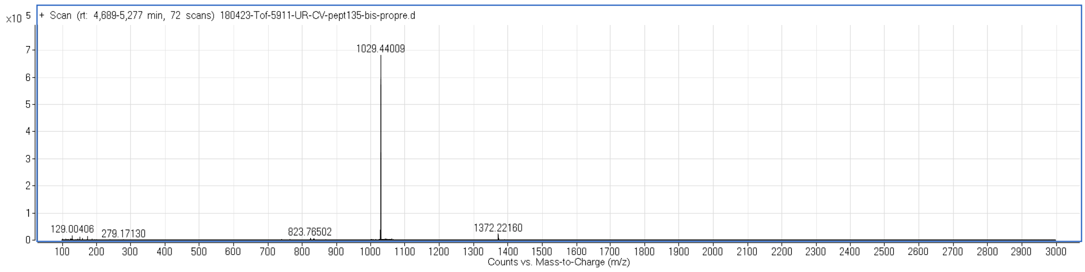

Supplementary Figure 86. LC-MS spectrum of peptide 25. Characterization of peptide **25** by LC-MS. The experiment was performed on UHPLC (Agilent 1290 Infinity) coupled to an ESI -MS Tof (Agilent 6230 ESI) with the dual-ESI source operated in positive ion mode. (Gradient: 10-100%; CH_3CN 0.1 % Formic acid in H_2O 0.1% formic acid, 6 min, C18).

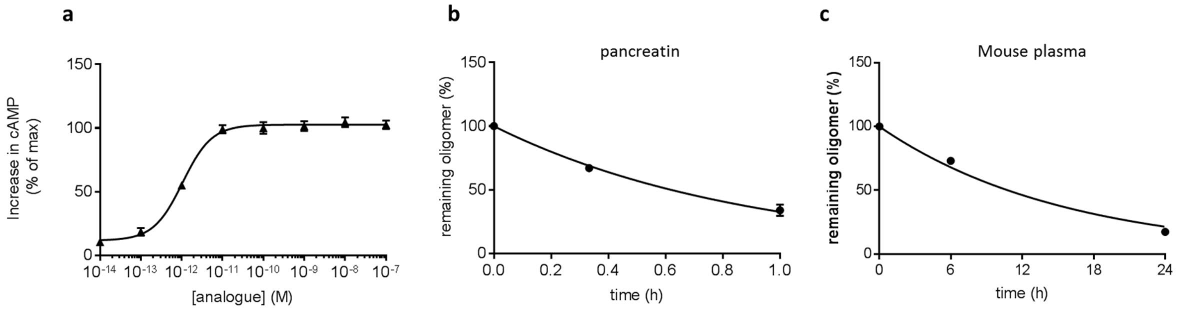

Supplementary Figure 87. In vitro pharmacology characterization. (a) Concentration-response curve (for EC50 determination of oligomer **25**. Evaluation of the agonist activity at the human GLP-1 receptor exogenously expressed in HEK293T cells, was determined by measuring their effects on cAMP production using the HTRF detection method. The data are mean ±SEM of a typical experiment performed 4 times. (b) Enzymatic degradation (pancreatin). The data are mean ±SEM of a typical experiment performed 3 times. (c) Mouse plasma degradation. The data are mean ±SEM of a typical experiment performed 3 times. Source data are provided as a Source Data file ((a): Supp Fig. 5 tab, (b): Fig. 6b tab, (c): Supp Fig. 3 tab).

Supplementary Methods

In vitro pharmacology (EC_{50}) – second method

Evaluation of the agonist activity of hybrid **24** and semaglutide (**25**) at the human GLP-1 receptor exogenously expressed in HEK293T cells (Multispan inc., lot#.DC1267-062017), was determined by measuring their effects on cAMP production using the HTRF detection method (performed by UREkA s.a.r.l.). The cells were suspended in cell culture media (DMEM 1X + GlutaMAX (Gibco 31966-021)) complemented with FBS 10% (Sigma Aldrich F7524), Pen/Strep 1% (Sigma Aldrich P4333) and 500 µM IBMX, then distributed in 384-well microplates at a density of $1.0x10^4$ cells/well (35 µL). Stock solutions of the compounds were prepared at a concentration of 1 mM in DMSO. Then, compounds to be tested were diluted in assay buffer and a 35 µL aliquot transferred to the plate containing the cells to reach final assay concentrations in the range 10^{-14} - 10^{-7} M. The cells were incubated for 15 min at 5% CO_2 at 37°C and then lysed. Fluorescence donor (anti-cAMP antibody labeled with europium cryptate) and the fluorescence acceptor (D2-labeled cAMP) were then added to the mixture of lysed cells. After 120 min at room temperature, a microplate reader (F500 Tecan) was used to measure the fluorescence transfer at λ_{ex} = 337 nm and λ_{em} = 620 nm and 665 nm. The ratio of the signal measured at 665 nm on signal measured at 620 nm is used to determine the cAMP concentration. The results are given as a percent of the control response to 10 nM Forskolin. The standard reference agonist is GLP-1-G^2-NH_2 (peptide **1**), which is tested in each experiment at several concentrations to generate a concentration-response curve from which its EC_{50} value is calculated using GraphPad Prism.

Synthesis of monomers (N_3-A^u-OSu) [1] and (N_3-I^u-OSu) [2]

Boc-L-AA-OH was dissolved in THF under N_2 at -10°C. 4-Methymorpholine (1.10 eq.) was added. Isobutyl chloroformate (1.05 eq.) dissolved in THF (10 mL) was added dropwise and the mixture was stirred 45min at -10°C. The insoluble were filtered.

Sodium borohydride (2.00 eq.) was dissolved in H_2O (4mL) and the previous filtrate was added dropwise at 0°C. The mixture was stirred overnight at room temperature.

THF was evaporated. The compound was dissolved in EtOAc, washed with $KHSO_4$ (1M), $NaHCO_3$ (sat) and brine (sat), dried with $MgSO_4$ and concentrated.

Previous compound was dissolved in anhydrous THF at 0°C and triphenylphosphine (1.20 eq.) and Phtalimide (1.20 eq.) were added. DIAD (1.20 eq.) was added dropwise at 0°C and the mixture was stirred 48h at RT.The solvent was evaporated and the compound was purified by flash column chromatography on silica gel. Eluent Cyclohexane/EtOAc 90:10 and then 80:20.

Previous compound was dissolved in Trifluoroacetic acid (30 mL) at 0°C and stirred 2h. TFA was evaporated and coevaporated with cyclohexane.

The previous TFA salt was dissolved in CH_3CN/H_2O (50:50). Potassium carbonate (1.00 eq.), Copper(II) sulfate pentahydrate (0.01 eq.) and imidazolium salt (1.20 eq.) were added. The mixture was stirred 1h30 at RT. EtOAc was added and the phase were separated. Aqueous phase was extracted with EtOAc. Organic phases were combined and washed with $KHSO_4$ and brine, dried with $MgSO_4$ and concentrated. The compound was purified by flash chromatography on silica gel (liquid loading). Eluent Cyclo/EtOAc 90:10.

Previous compound was dissolved in MeOH (15 ml) with DCM (4 ml). Hydrazine monohydrated (3.00 eq.) was added and the mixture was stirred at 70°C for 3h. The white precipitate was filtrated and washed with EtOAc. The MeOH was evaporated. The compound was dissolved in EtOAc and washed with water and brine, dried with MgSO4. The compound was used as is in the next step without evaporation.

N,N'-Disuccinimidyl carbonate (1.00 eq.) was suspended in EtOAc (20 mL) at 0°C. Previous compound dissolved in EtOAc (150 mL) was added dropwise at 0°C and the mixture was stirred 2h at room

temperature. The solvent was half evaporated. The organic phase was washed with $KHSO_4$ (1M), and brine (sat), dried with $MgSO_4$ and concentrated. The mixture was triturated in hexane to afford the desired monomer.

Characterization of monomer (N_3-A^u-OSu)[1]

(S)-2,5-dioxopyrrolidin-1-yl (2-azidopropyl)carbamate: **Melting point** (*M.p.*) 83-85°C, **^{1}H NMR** ($CDCl_3$, 300 MHz) δ 5.58 (bt, J = 6.0, 1H), 3.81 – 3.71 (m, 1H), 3.41 (ddd, J = 13.9, 7.0, 4.0, 1H), 3.14 (ddd, J = 13.7, 7.9, 5.4, 1H), 2.84 (s, 4H), 1.32 (d, J = 6.6, 3H**); ^{13}C NMR** ($CDCl_3$, 75 MHz) δ 169.90, 151.69, 77.51, 77.29, 77.09, 76.66, 56.96, 46.57, 25.50, 16.66; **HRMS** (*m/z*) cald for $C_8H_{11}N_5O_4Cl$ $[M+Cl]^-$ 276.0500, found 276.0490.

Characterization of monomer (N_3-I^u-OSu)[2]

2,5-dioxopyrrolidin-1-yl ((2S,3R)-2-azido-3-methylpentyl)carbamate: **^{1}H NMR** ($CDCl_3$, 300 MHz) δ: 5.52 (bs, 1H), 3.62 – 3.48 (m, 2H), 3.21 – 3.07 (m, 1H), 2.87 (s, 4H), 1.80 – 1.67 (m, 1H), 1.64-1.51 (m, 1H), 1.39-1.22 (m, 1H), 1.01 (d, J = 6.8 Hz, 1H), 0.98 (t, J = .4 Hz, 3H); **^{13}C NMR** ($CDCl_3$, 75 MHz) δ:169.84,151.61, 66.62, 42.96, 37.19, 25.48, 15.06, 11.38.

Synthesis of monomers (N_3-Y^u(*t*Bu)-OSu)[1] and (N_3-E^u(*t*Bu)-OSu)[3]

Fmoc-L-AA-OH was dissolved in THF under N_2 at -10°C. 4-Methymorpholine (1.10 eq.) was added. Isobutyl chloroformate (1.05 eq.) dissolved in THF (10 mL) was added dropwise and the mixture was stirred 45 min at -10°C. The insoluble were filtered.
Sodium borohydride (2.00 eq.) was dissolved in H_2O (4mL) and the previous filtrate was added dropwise at 0°C. The mixture was stirred overnight at room temperature.
THF was evaporated. The compound was dissolved in EtOAc, washed with $KHSO_4$ (1M), $NaHCO_3$ (sat) and brine (sat), dried with $MgSO_4$ and concentrated
The previous compound was dissolved in dioxane/Piperidine (80:20). The mixture was stirred 3h at RT. The dioxane was evaporated and the compound was purified by flash column chromatography on silica gel. Eluent Cyclohexane/EtOAc 70:30; 50:50; 30:70; 0:100 and then EtOAc/MeOH 80:20
Previous compound was dissolved in CH_3CN/H_2O (50:50). Potassium carbonate (1.00 eq.), Copper(II) sulfate pentahydrate (0.01 eq.) and imidazolium salt (1.20 eq.) were added. The mixture was stirred 1h30 at RT. EtOAc was added and the phase were separated. Aqueous phase was extracted with EtOAc. Organic phases were combined and washed with $KHSO_4$ and brine, dried with $MgSO_4$ and concentrated. The compound was purified by flash chromatography on silica gel (liquid loading). Eluent Cyclo/EtOAc 90:10 and then 80:20.
Previous compound was dissolved in anhydrous THF at 0°C and triphenylphosphine (1.20 eq.) and Phtalimide (1.20 eq.) were added. DIAD (1.20 eq.) was added dropwise at 0°C and the mixture was stirred 48h at RT.The solvent was evaporated and the compound was purified by flash column chromatography on silica gel. Eluent Cyclohexane/EtOAc 90:10 and then 80:20.
Previous compound was dissolved in MeOH (15 ml) with DCM (4 ml). Hydrazine monohydrated (3.00 eq.) was added and the mixture was stirred at 70°C for 3h. The white precipitate was filtrated and washed with EtOAc. The MeOH was evaporated. The compound was dissolved in EtOAc and washed with water and brine, dried with $MgSO_4$. The compound was used as is in the next step without evaporation.
N,N'-Disuccinimidyl carbonate (1.00 eq.) was suspended in EtOAc (20 mL) at 0°C. Previous compound dissolved in EtOAc (150 mL) was added dropwise at 0°C and the mixture was stirred 2h at room temperature.The solvent was half evaporated. The organic phase was washed with $KHSO_4$ (1M), and brine (sat), dried with $MgSO_4$ and concentrated. The mixture was triturated in hexane to afford the desired monomer.

Characterization of monomer (N_3-Y^u(*t*Bu)-OSu)[1]

(S)-2,5-dioxopyrrolidin-1-yl (2-azido-3-(4-(tert-butoxy)phenyl)propyl)carbamate:.**Melting point** (*M.p.*) 106-108 °C; **^{1}H NMR** ($CDCl_3$, 300 MHz) δ 7.13 (d, J=8.5, 2H), 6.95 (d, J=8.5, 2H), 5.51 (t, J= 5, 1H), 3.80 (m, 1H), 3.47 (m, 1H), 3.15 (m, 1H), 2.87 (d, J=6, 1H), 2.83 (bp, 5H), 1.33 (s, 9H); **^{13}C NMR** ($CDCl_3$, 75 MHz) δ 169.88, 154.64, 151.68, 131.15, 129.90, 124.71, 78.68, 63.28, 45.06, 38.03, 28.93, 25.58. **HRMS** (*m/z*) cald for $C_{18}H_{23}N_5O_5Na$ $[M+Na]^+$ 412.1597, found 412.1607.

Characterization of monomer (N_3-E^u(*t*Bu)-OSu)[3]

(S)-tert-butyl 4-azido-5-((((2,5-dioxopyrrolidin-1-yl)oxy)carbonyl)amino) pentanoate: **^{1}H NMR** ($CDCl_3$, 300 MHz) δ: 5.63 (bs, 1H), 3.74 – 3.66 (m, 1H), 3.46 (ddd, J= 14.2, 6.5, 4.5 Hz, 1H), 3.34 – 3.21 m, 1H), 2.86 (s, 4H), 2.43 (td, J= 7.1, 1.9 Hz, 2H), 2.01 – 1.78 (m, 2H), 1.49 (s, 9H), 1.23 (d, J= 6.2 Hz, H); **^{13}C NMR** ($CDCl_3$, 75 MHz) δ: 171.97, 169.73, 151.67, 81.08, 61.06, 45.02, 31.27, 28.08, 26.84, 5.48; **HRMS** (m/z): calcd for $C_{14}H_{22}N_5O_6$$[M+H]^+$ 356.1572, found 356.1572

Synthesis and characterization of Monomer M1 (N_3-$F^{u\alpha}$-OSu)

Boc-D-Phe-OH (6.00 g, 22.6 mmol) was dissolved in THF (100 mL) under N_2 at -10°C. 4-Methymorpholine (2.74 mL, 24.9 mmol) was added. Isobutyl chloroformate (2.93 mL, 22.6 mmol) dissolved in THF (10 mL) was added dropwise and the mixture was stirred 45min at -10°C. The insoluble were filtered.

Sodium borohydride (1.71 g, 45.2 mmol) was dissolved in H_2O (4mL) and the previous filtrate was added dropwise at 0°C. The mixture was stirred overnight at room temperature.

THF was evaporated. The compound was dissolved in EtOAc, washed with $KHSO_4$ (1M), $NaHCO_3$ (sat) and brine (sat), dried with $MgSO_4$ and concentrated.

I1 (5.16 g, 20.5 mmol) was dissolved in dry DCM (150 mL) under N_2 at 0°C. Triethylamine (2.08 g, 20.5 mmol) was added. Methanesulfonyl chloride (2.35 g, 20.5 mmol) was added dropwise at 0°C and the mixture was stirred 2h at 0°C.

Organic phase was washed with $KHSO_4$ (1M), $NaHCO_3$ (sat) and brine (sat), dried with $MgSO_4$ and concentrated.

I2 (6.44 g, 19.6 mmol) was dissolved in DMF (60.0 mL) and Sodium azide (5.08 g, 78.2 mmol) was added with 10mL of DMF. The mixture was stirred overnight at 80°C.

The mixture was cooled down to room temperature. 50 mL of H_2O and 100mL of EtOAc were added. The aqueouse phase was extracted twice with EtOAc. The organics phase were combined, washed with brine (sat), dried with $MgSO_4$ and concentrated. The compound was purified by flash column chromatography on silica gel. Eluent Cyclohexane/EtOAc 100:0 to 85:15.

I3 (3.40 g, 12.3 mmol) was dissolved in Trifluoroacetic acid (30 mL) at 0°C and stirred 45 min. The TFA was evaporated and coevaporated with cyclohexane.

The TFA salt (**I4**) was dissolved in anhydrous DCM (100 mL) under N_2 and cooled to 0°C. N,N-Diisopropylethylamine (2.15 mL, 12.3 mmol)and N,N'-Disuccinimidyl carbonate (3.16 g, 12.3 mmol) were added and the mixture was stirred 3h at room temperature under N_2.

The organic phase was washed with $KHSO_4$ (1M), and brine (sat), dried with $MgSO_4$ and concentrated. The compound was triturated in hexane to afford the monomer **M1** as a white powder (3.60 g) with a total yield of 50%.

Melting point (*M.p.*) 112–114°C; **^{1}H NMR** ($CDCl_3$, 300 MHz) δ: 7.40-7.20 (m, 5H), 5.86 (d, *J*= 8.1 Hz, 1H), 4.00 (m, 1H), 3.57-3.38 (ddd, *J*= 4.4, 4.6, 12.5 Hz, 2H),3.06-2.86 (ddd, *J*= 6.0, 8.4, 13.7 Hz, 2H), 2.84 (s, 1H) ; **^{13}C NMR** (75 MHz, $CDCl_3$) δ: 169.86, 150.92, 136.21, 129.32, 128.92, 127.13, 53.09, 52.27, 37.62, 25.48; **HRMS** (*m/z*) calcd for $C_{14}H_{15}N_5O_4Na^+$ $[M + Na]^+$ 340.1016, found 340.1004.

Synthesis and characterization of Monomer M2 (N_3-$D^{u\alpha}$(*t*Bu)-OSu)

Fmoc-D-Asp(OtBu)-OH (5.00 g, 11.9 mmol) was dissolved in dry THF (in 500 mL double neck flask) at (-10°C, ice + salt bath) under N_2. 4-Methymorpholine (1.45 mL, 13.1 mmol) was added

Isobutyl chloroformate (1.62 mL, 12.5 mmol) was added dropwise via an addition funnel. The mixture was stirred 45 min at -10°C.
The white precipitate was filtered and washed with THF.
Sodium borohydride (901 mg, 23.8 mmol) was dissolved in 5 ml of H_2O and the previous filtrate was added dropwise at 0°C.
The mixture was stirred 3h at RT.
THF was evaporated. The compound was solubilized in EtOAc and washed with $KHSO_4$ (1M), $NaHCO_3$ (sat) and brine (sat), dried with $MgSO_4$ and concentrated.
Triphenylphosphine (9.56 g, 36.5 mmol) and Imidazole (4.14 g, 60.8 mmol) were dissolved in anhydrous DCM under N_2. Iodine (9.25 g, 36.5 mmol) was added. **I5** (4.83 g, 12.2 mmol) previously dissolved in anhydrous DCM was added dropwise under N_2 and the mixture was stirred 3h at RT.
The solvent was evaporated and the compound was purified by flash column chromatography on silica gel. Eluent Cyclohexane/EtOAc 90:10
I6 (4.40 g, 8.67 mmol) was dissolved in DMF and Sodium azide (2.82 g, 43.4 mmol) was added. The mixture was stirred overnight at room temperature.
50 mL of water was added and the compound was extracted with EtOAc three times. Organic phases were combined, dried with $MgSO_4$ and concentrated.
The compound was purified by flash column chromatography on silica gel. Eluent Cyclohexane/EtOAc 90:10 ; 80:20
I7 (2.20 g, 5.21 mmol) was dissolved in THF and Piperidine (10.3 mL, 104 mmol) was added. The mixture was stirred over night at RT.
The THF was evaporated and the compound was purified by flash column chromatography on silica gel. Eluent Cyclohexane/EtOAc 70:30; 50:50; 30:70; 0:100 and then EtOAc/MeOH 80:20
N,N'-Disuccinimidyl carbonate (1.45 g, 5.55 mmol) was dissolved in EtOAc (20 mL) at 0°C. **I8** (1.00 g, 4.99 mmol) previously dissolved in EtOAc (100 mL) was added dropwise at 0°C and the mixture was stirred 2h at room temperature.
The solvent was half evaporated. The organic phase was washed with $KHSO_4$ (1M), and brine (sat), dried with $MgSO_4$ and concentrated. The mixture was triturated in hexane to afford the monomer **M2** as a white powder (1.37g) with a total yield of 33%.
Melting point (*M.p.*) 92–94°C; **^{1}H NMR** ($CDCl_3$, 300 MHz) δ: 6.34 (d, *J*= 8.46 Hz, 1H), 4.17-4.06 (m, 1H), 3.65-3.50 (m, 2H) 2.85 (s, 4H), 2.63 (d, *J*= 5.72 Hz, 2H), 1.49 (s, 9H) ; **^{13}C NMR** (75 MHz, $CDCl_3$) δ: 169.95, 169.76, 150.91, 82.22, 53.18, 48.87, 36.64, 28.01, 25.48; **HRMS** (*m/z*) calcd for $C_{13}H_{19}N_5O_6Na^+$ $[M + Na]^+$ 364.1228, found 364.1213.

General procedure A for the Synthesis of oligomers 1-23

Oligomers **1-23** were synthesized using the following general procedure

Sieber resin (≈ 160mg, loading 0.62mmol.g^{-1}) was swelled in DMF (3 mL) for 30 min. All steps were performed under microwave irradiation. The synthesis were conducted with microwave irradiation using the Liberty Blue™ microwave peptide synthesizer from CEM.

The *N*-Fmoc protecting group was removed with 20% piperidine in DMF (3 ml) with the standard liberty blue methods.[4]

N-Fmoc-α amino acid (5 eq. relative to the resin loading) were coupled with PyBOP (5 eq. relative to the resin loading) and DIEA (10 eq. relative to the resin loading) as coupling reagent using the standard liberty blue methods.[4]

Each activated monomer (3 eq. relative to the resin loading) was coupled twice using DIEA (10 eq. relative to the resin loading) under microwave irradiation (70°C, 50W, 20 min) in DMF (4 mL).

The reduction of the azido group was performed twice in a mixture of 1,4-dioxane/H_2O (7:3 v/v) (5 mL) with a 1M PMe_3 solution in THF (10 eq. relative to the resin loading) under microwave irradiation (50°C, 50W, 30 min).

After completion of the synthesis, the resin was transferred into a syringe with a frit, and washed three times with DMF, three times with CH_2Cl_2 and three times with Et_2O. Cleavage from the resin was performed using 95% TFA with 2.5% triisopropylsilane and 2.5% water (3 mL). After 2h the resin was filtered and discarded. Diethyl ether was added to precipitate the oligomer and the solid was triturated and filtrated.

Analytical RP-HPLC analyses were performed on a Dionex U3000SD using a Macherey-Nagel Nucleodur C18ec column (4 x 100 mm, 3 µm) at a flow rate of 1 mL.min^{-1} with UV detection at 200 nm. The mobile phase was composed of 0.1% (v/v) TFA-H_2O (Solvent A) and 0.1% (v/v) TFA-CH_3CN (solvent B).

Semi preparative purification of all compound was performed on a Dionex U3000SD using a Macherey-Nagel Nucleodur C18ec column (10 x 250 mm, 5 µm) at a flow rate of 4 mL.min^{-1} with UV detection at 200 nm. The mobile phase was composed of 0.1% (v/v) TFA-H_2O (Solvent A) and 0.1% (v/v) TFA-CH_3CN (solvent B).

LC-MS analyses were carried out on a UHPLC (Agilent 1290 Infinity) coupled to a ESI -MS Tof (Agilent 6230 ESI).

Electrospray ionization mass spectrometry (ESI-MS) experiments were performed on an Agilent 6560 DTIMS-Q-TOF spectrometer (Agilent Technologies, Santa Clara, CA), with the dual-ESI source operated in positive ion mode.

Procedure B for the synthesis of oligomer 24

N-Fmoc-Lys(Boc)-OH at position 20 was replaced by *N*-Fmoc-Lys(Alloc)-OH and *N*-Fmoc-His(Trt)-OH at position 1 was replaced by *N*-Boc-His(Boc)-OH.

The oligomer was synthesized using procedure **A**. Then the resin was transferred in a 10 mL syringe, 5 mL of DCM was added and the Alloc group was removed using $Pd(Ph_3)_4$ (30 mg, 0.25 equiv relative to the resin loading) and phenylsilane (135 µL, 1.1 equiv relative to the resin loading) at room temperature for 45 min. After filtration and washes (3x DCM), DCM (5 mL), Fmoc-OcO_2-OH (193 mg, 5 equiv), PyBop (260 mg, 5 equiv relative to the resin loading) and DIEA (93 µL, 5 equiv relative to the resin loading) were loaded on the resin and it was shaken for 2 hours at room temperature. After filtration and washes (3x DCM, 3x DMF) Fmoc group was removed with piperidine in DMF (20%), 2 times 20 min. The resin was washed with DMF (2x) and DCM (3x), then DCM (5 mL), Fmoc-OcO_2-OH (193 mg, 5 equiv relative to the resin loading), PyBop (260 mg, 5 equiv relative to the resin loading) and DIEA (93 µL, 5 equiv relative to the resin loading) were loaded on the resin and it was shaken for 2 hours at room temperature and again Fmoc group was removed with piperidine (20%) twice. The resin was washed with DMF (2x) and DCM (3x), then DCM (5 mL), *N*-Fmoc-Glu(OH)-O*t*Bu (222 mg, 5 equiv relative to the resin loading), PyBop (260 mg, 5 equiv relative to the resin loading), and DIEA (93 µL, 5 equiv relative to the resin loading) were loaded on the resin and it was shaken for 2 hours at room temperature. Fmoc group was removed with piperidine in DMF (20%), 2 times 20 min. The resin was washed with DMF (2x) and DCM (3x), then the 18-(ter-butoxy)18-oxooctadecanoic acid (111 mg, 3 equiv relative to the resin loading), ByBop (156 mg, 3 equiv relative to the resin loading) and DIEA (52 µL, 3 equiv relative to the resin loading) were loaded on the resin and it was shaken for 2 hours at

room temperature. The cleavage, the purification and the characterization were the same as procedure A.

Procedure C for the synthesis of semaglutide (25)

N-Fmoc-Lys(Boc)-OH at position 20 was replaced by *N*-Fmoc-Lys(Alloc)-OH and *N*-Fmoc-His(Trt)-OH at position 1 was replaced by *N*-Boc-His(Boc)-OH.

For oligomer 25, the first amino acid, *N*-Fmoc-Gly-OH was coupled on the resin with mixt anhydride (Novabiochem ® 2014/2015, 3.6 (Method 3-8)). *N*-Fmoc-Gly-OH (300 mg, 5 equiv relative to the resin loading) was dissolved in dry DCM (3 mL) under inert atmosphere. *N,N'*-Dicyclohexylcarbodiimide (103 mg, 2.5 equiv relative to the resin loading) was added and the mixture was stirred 20 min at 0°C then DCM was concentrated and DMF was added. The mixture was loaded on the swollen *Wang* resin (loading 0.51 mmol/g) with a catalytic amount of 4-Dimethylaminopyridine (DMAP). The resin was shaked for 2 hours at room temperature.

All the others amino acid were coupled using the procedure A. The cleavage, the purification and the characterization were the same as procedure A.

Characterization of H-HGEGTFTSDVSSYLEGQAAKEFIAWLVKGRG-NH_2 (1)

Peptide **1** has been synthesized using the general procedure **A** starting from sieber resin (160 mg, 0.1 mmol). The final product **1** was purified by semi-preparative HPLC. 6.1 mg was obtained (yield 1.8 %). **HPLC**: R_t= 5.29 min (10-100%; CH_3CN 0.1 % TFA in H_2O 0.1% TFA, 10 min, C18); **LC-MS** *(m/z 3340.71)*: 668.78 $[M+5H]^{5+}$, 835.75 $[M+4H]^{4+}$, 1114.36 $[M+3H]^{3+}$ 1671.10 $[M+2H]^{2+}$

Characterization of H-HGEGTFTS$D^{u\alpha}A^{u}A^{u}$YLEGQAAKEFIAWLVKGRG-NH_2 (2)

Oligomer **2** has been synthesized using the general procedure **A** starting from sieber resin (160 mg, 0.1 mmol). The final product **2** was purified by semi-preparative HPLC. 10.13 mg was obtained (yield 3.1 %). **HPLC**: R_t= 5.28 min (10-100% CH_3CN 0.1 % TFA in H_2O 0.1% TFA, 10 min, C18); **LC-MS** *(m/z 3296.70)*: 660.15 $[M+5H]^{5+}$, 825.19$[M+4H]^{4+}$, 1099.58 $[M+3H]^{3+}$, 1649.37 $[M+2H]^{2+}$

Characterization of H-HGEGTFTSD$A^{u}A^{u}A^{u}$LEGQAAKEFIAWLVKGRG-NH_2 (3)

Oligomer **3** has been synthesized using the general procedure **A** starting from sieber resin (160 mg, 0.1 mmol). The final product **3** was purified by semi-preparative HPLC. 28.4 mg was obtained (yield 8.9 %). **HPLC**: R_t= 5.25 min (10-100% CH_3CN 0.1 % TFA in H_2O 0.1% TFA, 10 min, C18); **ESI+** *(m/z 3204.61)*: 641.67 $[M+5H]^{5+}$, 802.13 $[M+4H]^{4+}$, 1069.27 $[M+3H]^{3+}$, 1603.27 $[M+2H]^{2+}$

Characterization of H-HGEGTFTSDV$A^{u}A^{u}A^{u}$EGQAAKEFIAWLVKGRG-NH_2 (4)

Oligomer **4** has been synthesized using the general procedure **A** starting from sieber resin (160 mg, 0.1 mmol). The final product **4** was purified by semi-preparative HPLC. 7.0 mg was obtained (yield 2.2 %). **HPLC**: R_t= 5.01 min (10-100% CH_3CN 0.1 % TFA in H_2O 0.1% TFA, 10 min, C18); **ESI+** *(m/z 3190.58)*: 639.13 $[M+5H]^{5+}$, 798.60 $[M+4H]^{4+}$, 1064.47 $[M+3H]^{3+}$, 1595.87 $[M+2H]^{2+}$

Characterization of H-HGEGTFTSDV$A^{u}Y^{u}A^{u}$EGQAAKEFIAWLVKGRG-NH_2 (5)

Oligomer **5** has been synthesized using the general procedure **A** starting from sieber resin (160 mg, 0.1 mmol). The final product **5** was purified by semi-preparative HPLC. 2.4 mg was obtained (yield 0.8 %).

HPLC: R_t= 5.08 min (10-100% CH_3CN 0.1 % TFA in H_2O 0.1% TFA, 10 min, C18); **LC-MS** *(m/z 3282.67)*: 657.36$[M+5H]^{5+}$, 821.45 $[M+4H]^{4+}$, 1094.93 $[M+3H]^{3+}$, 1641.89 $[M+2H]^{2+}$

Characterization of H-HGEGTFTSDVSA^{u}A^{u}A^{u}GQAAKEFIAWLVKGRG-NH_2 (6)

Oligomer **6** has been synthesized using the general procedure **A** starting from sieber resin (160 mg, 0.1 mmol). The final product **6** was purified by semi-preparative HPLC. 9.3 mg was obtained (yield 2.95 %). **HPLC**: R_t= 5.08 min (10-100% CH_3CN 0.1 % TFA in H_2O 0.1% TFA, 10 min, C18); **ESI+** *(m/z 3148.54)*: 630.67 $[M+5H]^{5+}$, 788.00 $[M+4H]^{4+}$, 1050.27 $[M+3H]^{3+}$, 1575.33 $[M+2H]^{2+}$

Characterization of H-HGEGTFTSDVSSA^{u}A^{u}A^{u}QAAKEFIAWLVKGRG-NH_2 (7)

Oligomer **7** has been synthesized using the general procedure **A** starting from sieber resin (160 mg, 0.1 mmol). The final product **7** was purified by semi-preparative HPLC. 11.88 mg was obtained (yield 3.7 %). **HPLC**: R_t= 5.13 min (10-100% CH_3CN 0.1 % TFA in H_2O 0.1% TFA, 10 min, C18); **ESI+** *(m/z 3178.57)*: 795.51 $[M+4H]^{4+}$, 1060.63 $[M+3H]^{3+}$, 1590.80 $[M+2H]^{2+}$

Characterization of H-HGEGTFTSDVSSYA^{u}A^{u}A^{u}AAKEFIAWLVKGRG-NH_2 (8)

Oligomer **8** has been synthesized using the general procedure **A** starting from sieber resin (160 mg, 0.1 mmol). The final product **8** was purified by semi-preparative HPLC. 3.22mg was obtained (yield 1.0 %). **HPLC**: R_t= 5.20 min (10-100% CH_3CN 0.1 % TFA in H_2O 0.1% TFA, 10 min, C18); **ESI+** *(m/z 3213.61)*: 643.60 $[M+5H]^{5+}$, 804.33 $[M+4H]^{4+}$, 1072.07 $[M+3H]^{3+}$, 1607.73$[M+2H]^{2+}$

Characterization of H-HGEGTFTSDVSSYY^{u}E^{u}A^{u}AAKEFIAWLVKGRG-NH_2 (9)

Oligomer **9** has been synthesized using the general procedure **A** starting from sieber resin (160 mg, 0.1 mmol). The final product **9** was purified by semi-preparative HPLC. 2.3 mg was obtained (yield 0.7 %). **HPLC**: R_t= 5.11 min (10-100% CH_3CN 0.1 % TFA in H_2O 0.1% TFA, 10 min, C18); **LC-MS** *(m/z 3363.74)*: 673.74 $[M+5H]^{5+}$, 841.68 $[M+4H]^{4+}$, 1121.90 $[M+3H]^{3+}$, 1682.85 $[M+2H]^{2+}$

Characterization of H-HGEGTFTSDVSSYLA^{u}A^{u}A^{u}AKEFIAWLVKGRG-NH_2 (10)

Oligomer **10** has been synthesized using the general procedure **A** starting from sieber resin (160 mg, 0.1 mmol). The final product **10** was purified by semi-preparative HPLC. 13.02 mg was obtained (yield 4.0 %). **HPLC**: R_t= 5.31 min (10-100% CH_3CN 0.1 % TFA in H_2O 0.1% TFA, 10 min, C18); **ESI+** *(m/z 3255.69)*: 814.80 $[M+4H]^{4+}$, 1086.33 $[M+3H]^{3+}$, 1628.33 $[M+2H]^{2+}$

Characterization of H-HGEGTFTSDVSSYLE^{u}A^{u}A^{u}AKEFIAWLVKGRG-NH_2 (11)

Oligomer **11** has been synthesized using the general procedure **A** starting from sieber resin (160 mg, 0.1 mmol). The final product **11** was purified by semi-preparative HPLC. 17.6 mg was obtained (yield 5.3 %). **HPLC**: R_t= 5.27 min (10-100% CH_3CN 0.1 % TFA in H_2O 0.1% TFA, 10 min, C18); **LC-MS** *(m/z 3313.73)*: 663.55 $[M+5H]^{5+}$, 829.19 $[M+4H]^{4+}$, 1105.25 $[M+3H]^{3+}$

Characterization of H-HGEGTFTSDVSSYLEA^{u}A^{u}A^{u}KEFIAWLVKGRG-NH_2 (12)

Oligomer **12** has been synthesized using the general procedure **A** starting from sieber resin (160 mg, 0.1 mmol). The final product **12** was purified by semi-preparative HPLC. 10.2 mg was obtained (yield 3.1 %). **HPLC**: R_t= 5.19 min (10-100% CH_3CN 0.1 % TFA in H_2O 0.1% TFA, 10 min, C18); **ESI+** *(m/z 3313.73)*: 829.53 $[M+4H]^{4+}$, 1105.40 $[M+3H]^{3+}$, 1657.87$[M+2H]^{2+}$

Characterization of H-HGEGTFTSDVSSYLEG$A^uA^uA^u$EFIAWLVKGRG-NH_2 (13)

Oligomer **13** has been synthesized using the general procedure **A** starting from sieber resin (160 mg, 0.1 mmol). The final product **13** was purified by semi-preparative HPLC. 10.98 mg was obtained (yield 3.4 %). **HPLC**: R_t= 5.45 min (10-100% CH_3CN 0.1 % TFA in H_2O 0.1% TFA, 10 min, C18); **ESI+** *(m/z 3242.61)*: 811.53 $[M+4H]^{4+}$, 1081.73 $[M+3H]^{3+}$, 1621.87 $[M+2H]^{2+}$

Characterization of H-HGEGTFTSDVSSYLEGQ$A^uA^uA^u$FIAWLVKGRG-NH_2 (14)

Oligomer **14** has been synthesized using the general procedure **A** starting from sieber resin (160 mg, 0.1 mmol). The final product **14** was purified by semi-preparative HPLC. 6.0 mg was obtained (yield 1.85 %). **HPLC**: R_t= 5.48 min (10-100% CH_3CN 0.1 % TFA in H_2O 0.1% TFA, 10 min, C18); **LC-MS** *(m/z 3241.62)*: 649.14 $[M+5H]^{5+}$, 811.17$[M+4H]^{4+}$, 1081.23 $[M+3H]^{3+}$, 1621.33$[M+2H]^{2+}$

Characterization of H-HGEGTFTSDVSSYLEGQA$A^uA^uA^u$IAWLVKGRG-NH_2 (15)

Oligomer **15** has been synthesized using the general procedure **A** starting from sieber resin (160 mg, 0.1 mmol). The final product **15** was purified by semi-preparative HPLC. 8.4 mg was obtained (yield 2.6 %). **HPLC**: R_t= 5.05 min (10-100% CH_3CN 0.1 % TFA in H_2O 0.1% TFA, 10 min, C18); **ESI+** *(m/z 3165.52)*: 792.27 $[M+4H]^{4+}$, 1056.00 $[M+3H]^{3+}$, 1583.53 $[M+2H]^{2+}$

Characterization of H-HGEGTFTSDVSSYLEGQAAKE$F^{u\alpha}I^uA^u$LVKGRG-NH_2 (16)

Oligomer **16** has been synthesized using the general procedure **A** starting from sieber resin (160 mg, 0.1 mmol). The final product **16** was purified by semi-preparative HPLC. 4.8 mg was obtained (yield 1.5 %). **HPLC**: R_t= 5.07 min (10-100% CH_3CN 0.1 % TFA in H_2O 0.1% TFA, 10 min, C18); **ESI+** *(m/z 3241.62)*: 811.60 $[M+4H]^{4+}$, 1081.53 $[M+3H]^{3+}$, 1621.80 $[M+2H]^{2+}$

Characterization of H-HGEGTFTSDVSSYLEGQAAKEF$A^uA^uA^u$VKGRG-NH2 (17)

Oligomer **17** has been synthesized using the general procedure **A** starting from sieber resin (160 mg, 0.1 mmol). The final product **17** was purified by semi-preparative HPLC. 10.3 mg was obtained (yield 3.3 %). **HPLC**: R_t= 4.52 min (10-100% CH_3CN 0.1 % TFA in H_2O 0.1% TFA, 10 min, C18); **ESI+** *(m/z 3157.46)*: 632.47 $[M+5H]^{5+}$, 790.20 $[M+4H]^{4+}$, 1053.27 $[M+3H]^{3+}$, 1579.73 $[M+2H]^{2+}$

Characterization of H-HGEGTFTSDVSSYLEGQAAKEFI$A^uA^uA^u$KGRG-NH_2 (18)

Oligomer **18** has been synthesized using the general procedure **A** starting from sieber resin (160 mg, 0.1 mmol). The final product **18** was purified by semi-preparative HPLC. 10.0 mg was obtained (yield 3.2 %). **HPLC**: R_t= 4.60 min (10-100% CH_3CN 0.1 % TFA in H_2O 0.1% TFA, 10 min, C18); **ESI+** *(m/z 3171.48)*: 635.07 $[M+5H]^{5+}$, 794.00 $[M+4H]^{4+}$, 1058.20 $[M+3H]^{3+}$, 1586.73 $[M+2H]^{2+}$

Characterization of H-HGEGTFTSDVSSYLEGQAAKEFIA$A^uA^uA^u$GRG-NH_2 (19)

Oligomer **19** has been synthesized using the general procedure **A** starting from sieber resin (160 mg, 0.1 mmol). The final product **19** was purified by semi-preparative HPLC. 2.3 mg was obtained (yield 0.7 %). **HPLC**: R_t= 4.68 min (10-100% CH_3CN 0.1 % TFA in H_2O 0.1% TFA, 10 min, C18); **LC-MS** *(m/z 3114.39)*: 623.72 $[M+5H]^{5+}$, 779.39 $[M+4H]^{4+}$, 1038.86 $[M+3H]^{3+}$, 1557.78 $[M+2H]^{2+}$

Characterization of H-HGEGTFTSDVSSYLEGQAAKEFIAWA^{u}A^{u}A^{u}RG-NH_2 (20)

Oligomer **20** has been synthesized using the general procedure **A** starting from sieber resin (160 mg, 0.1 mmol). The final product **20** was purified by semi-preparative HPLC. 2.05 mg was obtained (yield 0.6 %). **HPLC**: R_t= 5.21 min (10-100% CH_3CN 0.1 % TFA in H_2O 0.1% TFA, 10 min, C18); **LC-MS** *(m/z 3243.55)*: 649.59 $[M+5H]^{5+}$, 811.73 $[M+4H]^{4+}$, 1081.95 $[M+3H]^{3+}$, 1622.41 $[M+2H]^{2+}$

Characterization of H-HGEGTFTSDVSSYLEGQAAKEFIAWLA^{u}A^{u}A^{u}G-NH_2 (21)

Oligomer **21** has been synthesized using the general procedure **A** starting from sieber resin (160 mg, 0.1 mmol). The final product **21** was purified by semi-preparative HPLC. 2.7 mg was obtained (yield 0.8 %). **HPLC**: R_t= 5.81 min (10-100% CH_3CN 0.1 % TFA in H_2O 0.1% TFA, 10 min, C18); **LC-MS** *(m/z 3200.52)*: 800.97 $[M+4H]^{4+}$, 1067.95 $[M+3H]^{3+}$, 1600.89 $[M+2H]^{2+}$

Characterization of H-HGEGTFTSDVSSYLEGQAAKEFIAWLVA^{u}A^{u}A^{u}-NH_2 (22)

Oligomer **22** has been synthesized using the general procedure **A** starting from sieber resin (160 mg, 0.1 mmol). The final product **22** was purified by semi-preparative HPLC. 2.2 mg was obtained (yield 0.7 %). **HPLC**: R_t= 6.45 min (10-100% CH_3CN 0.1 % TFA in H_2O 0.1% TFA, 10 min, C18); **LC-MS** *(m/z 3242.60)*: 649.59 $[M+5H]^{5+}$, 811.48 $[M+4H]^{4+}$, 1081.63 $[M+3H]^{3+}$, 1621.91 $[M+2H]^{2+}$

Characterization of H-HGEGTFTSDVSSYY^{u}E^{u}A^{u}A^{u}A^{u}A^{u}FIAWLVKGRG-NH_2 (23)

Oligomer **23** has been synthesized using the general procedure **A** starting from sieber resin (160 mg, 0.1 mmol). The final product **23** was purified by semi-preparative HPLC. 5.5 mg was obtained (yield 1.7 %). **HPLC**: R_t= 5.57 min (10-100% CH_3CN 0.1 % TFA in H_2O 0.1% TFA, 10 min, C18); **LC-MS** *(m/z 3264.66)*: 653.69 $[M+5H]^{5+}$, 816.87 $[M+4H]^{4+}$, 1088.84 $[M+3H]^{3+}$

Characterization of H-HAibEGTFTSDVSSYLEGQAAK(2xOEG-γE-C18 diacid)EFIAWLVA^{u}A^{u}A^{u}-NH_2 (24)

2xOEG-γE-C18 diacid =

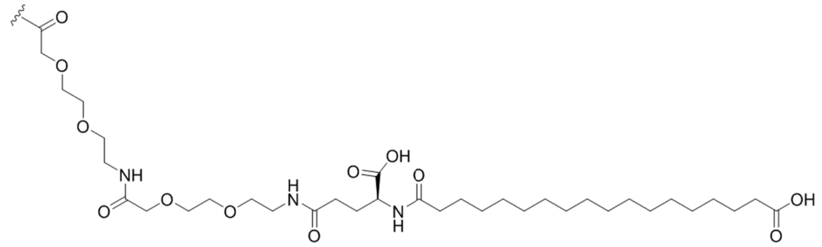

Oligomer **24** has been synthesized using the general procedure **B** starting from rink resin (196 mg, 0.1 mmol). The final product **24** was purified by semi-preparative HPLC. 1.45 mg was obtained (yield 0.4 %). **HPLC**: R_t= 8.27 min (10-100% CH_3CN 0.1 % TFA in H_2O 0.1% TFA, 10 min, C18); **LC-MS** *(m/z 3986.54)*: 997.67 $[M+4H]^{4+}$, 1329.87 $[M+3H]^{3+}$, 1994.26 $[M+2H]^{2+}$

Characterization of H-HAibEGTFTSDVSSYLEGQAAK(2xOEG-γE-C18 diacid)EFIAWLVRGRG-OH (25 Semaglutide)

2xOEG-γE-C18 diacid =

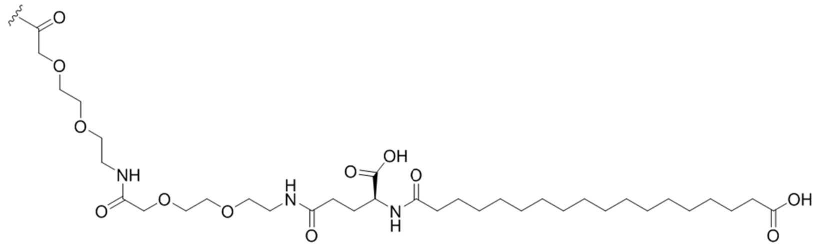

Peptide **25** has been synthesized using the general procedure **C** starting from wang resin (220 mg, 0.1 mmol). The final product **25** was purified by semi-preparative HPLC. 2.2 mg was obtained (yield 0.5 %). **HPLC**: R_t= 6.13 min (10-100% CH_3CN 0.1 % TFA in H_2O 0.1% TFA, 10 min, C18); **LC-MS** *(m/z 4113.60)*: 823.76 $[M+5H]^{5+}$, 1029.44 $[M+4H]^{4+}$, 1372.22 $[M+3H]^{3+}$

Supplementary References

1. Douat-Casassus, C., Pulka, K., Claudon, P. & Guichard, G. Microwave-Enhanced Solid-Phase Synthesis of N,N'-Linked Aliphatic Oligoureas and Related Hybrids. *Org. Lett.* **14**, 3130–3133 (2012)
2. Collie, G. W., Pulka-Ziach, K. & Guichard, G. Surfactant-facilitated crystallisation of water-soluble foldamers. *Chem. Sci.*, **7**, 3377-3383 (2016)
3. Collie, G. W. *et al.* Shaping quaternary assemblies of water-soluble non-peptide helical foldamers by sequence manipulation. *Nat. Chem*. **7**, 871–878 (2015)
4. Collins, J.M., Porter, K. A., Singh, S. K. & Vanier, G. S. High-Efficiency Solid Phase Peptide Synthesis (HE-SPPS). *Org. Lett.* **16**, 940−943 (2014)
