# Supplementary material for: Peptide-oligourea hybrids analogue of GLP-1 with improved action in vivo
Source: Nat Commun. 2019 Feb 25;10:924. doi: 10.1038/s41467-019-08793-y (PMC6389962; doi:10.1038/s41467-019-08793-y)
Supplement: Supplementary file 3 — Reporting Summary [file 41467_2019_8793_MOESM3_ESM.pdf]

nature research

Corresponding author(s): *Double-blind peer review submissions: write DBPR and your manuscript number here instead of author names.*

Last updated by author(s): *YYYY-MM-DD*

Reporting Summary

Nature Research wishes to improve the reproducibility of the work that we publish. This form provides structure for consistency and transparency in reporting. For further information on Nature Research policies, see Authors & Referees and the Editorial Policy Checklist.

Statistics

For all statistical analyses, confirm that the following items are present in the figure legend, table legend, main text, or Methods section.

n/a	Confirmed	
☐	☒	The exact sample size (*n*) for each experimental group/condition, given as a discrete number and unit of measurement
☐	☒	A statement on whether measurements were taken from distinct samples or whether the same sample was measured repeatedly
☐	☒	The statistical test(s) used AND whether they are one- or two-sided<br>*Only common tests should be described solely by name; describe more complex techniques in the Methods section.*
☒	☐	A description of all covariates tested
☒	☐	A description of any assumptions or corrections, such as tests of normality and adjustment for multiple comparisons
☒	☐	A full description of the statistical parameters including central tendency (e.g. means) or other basic estimates (e.g. regression coefficient) AND variation (e.g. standard deviation) or associated estimates of uncertainty (e.g. confidence intervals)
☒	☐	For null hypothesis testing, the test statistic (e.g. *F*, *t*, *r*) with confidence intervals, effect sizes, degrees of freedom and *P* value noted<br>*Give P values as exact values whenever suitable.*
☒	☐	For Bayesian analysis, information on the choice of priors and Markov chain Monte Carlo settings
☒	☐	For hierarchical and complex designs, identification of the appropriate level for tests and full reporting of outcomes
☒	☐	Estimates of effect sizes (e.g. Cohen's *d*, Pearson's *r*), indicating how they were calculated

Our web collection on statistics for biologists contains articles on many of the points above.

Software and code

Policy information about availability of computer code

Data collection	No software was used.
Data analysis	GraphPad Prism 7.0

For manuscripts utilizing custom algorithms or software that are central to the research but not yet described in published literature, software must be made available to editors/reviewers. We strongly encourage code deposition in a community repository (e.g. GitHub). See the Nature Research guidelines for submitting code & software for further information.

Data

Policy information about availability of data

All manuscripts must include a data availability statement. This statement should provide the following information, where applicable:

- Accession codes, unique identifiers, or web links for publicly available datasets
- A list of figures that have associated raw data
- A description of any restrictions on data availability

Data supporting findings of this manuscript are available from the corresponding authors upon reasonable request. Additional information on the synthesis of the GLP-1 analogues and their characterizations is available in the Supplementary data (Supplementary Methods and Supplementary Figs 7-87). The source data underlying Figs 2-7 and supplementary Fig 1-6 are provided as a Source Data file.

Field-specific reporting

Please select the one below that is the best fit for your research. If you are not sure, read the appropriate sections before making your selection.

☒ Life sciences ☐ Behavioural & social sciences ☐ Ecological, evolutionary & environmental sciences

For a reference copy of the document with all sections, see nature.com/documents/nr-reporting-summary-flat.pdf

Life sciences study design

All studies must disclose on these points even when the disclosure is negative.

Sample size	Sample sizes were based on previous publications showing that n=3 was sufficient to obtain statistical significance. Whenever possible, we tried to increase this number depending on the availability of the mice and cost of the studies, ranging from n=3 to n=6.
Data exclusions	Data > ± 2SD were considered as outliers and not considered for analysis. In addition, data were excluded when involuntary deviations of the method were done.
Replication	All replications were reported in the manuscript and the supplementary data and were successful.
Randomization	Blood glucose, IPGTT and PK studies: healthy mice (male C57BL/6J mice) were randomized into groups according to their body weight.<br>Blood glucose on db/db mice: db/db mice were randomized in 5 homogenous groups (n = 5 per group) according to their blood glucose, HOMA-IR and body weight.
Blinding	The investigators (Physiogenex and TechMedILL) were blinded to test items. Group allocations were done by Physiogenex and TechMedILL.<br>Blinding to group allocation was unnecessessary as Physiogenex and TechMedILL were independant CROs with limited knowledge on the nature of the test items.

Reporting for specific materials, systems and methods

We require information from authors about some types of materials, experimental systems and methods used in many studies. Here, indicate whether each material, system or method listed is relevant to your study. If you are not sure if a list item applies to your research, read the appropriate section before selecting a response.

Materials & experimental systems

n/a	Involved in the study	
☒	☐	Antibodies
☐	☒	Eukaryotic cell lines
☒	☐	Palaeontology
☐	☒	Animals and other organisms
☒	☐	Human research participants
☒	☐	Clinical data

Methods

n/a	Involved in the study	
☒	☐	ChIP-seq
☒	☐	Flow cytometry
☒	☐	MRI-based neuroimaging

Eukaryotic cell lines

Policy information about cell lines

Cell line source(s)	betaTC6 cells: Cerep S.A., catalog 2015, ref. 2181<br>HEK293T cells: Multispan Inc., lot#.DC1267-062017
Authentication	Cells were commercially available and presumably authentified by the provider.
Mycoplasma contamination	Cells were commercially available and presumably tested for mycoplasma contamination by the provider.
Commonly misidentified lines<br>(See ICLAC register)	No commonly misidentified cell lines were used.

Animals and other organisms

Policy information about studies involving animals; ARRIVE guidelines recommended for reporting animal research

Laboratory animals	Mice (male C57BL/6J mice (Charles River laboratories) 8 weeks old, 20-25 g)<br>Mice (male C57BL/6J mice (Charles River laboratories) 6 weeks old, 20-25 g)<br>Mice (male db/db mice (Charles River laboratories) 7 weeks old, 40 g)<br>Mice (male C57BL/6J mice (Janvier Labs, France) 9 weeks old, 20-25 g)

Wild animals	The study did not involve wild animals.
Field-collected samples	The study did not involve samples collected from the field.
Ethics oversight	.All animal protocols done by Physiogenex S.A.S were reviewed and approved by the local (Comité régional d'éthique de Midi-Pyrénées) and national (Ministère de l'Enseignement Supérieur et de la Recherche) ethics committees (protocol number 05049-06).<br>All animal protocols done by TechMedILL service were reviewed and approved by the agriculture ministry regulating animal research in France (Ethics regional committee for animal experimentation Strasbourg, APAFIS 1341#2015080309399690)

Note that full information on the approval of the study protocol must also be provided in the manuscript.
